# Mutation landscape in Chinese nodal diffuse large B-cell lymphoma by targeted next generation sequencing and their relationship with clinicopathological characteristics

**DOI:** 10.1186/s12920-024-01866-y

**Published:** 2024-04-13

**Authors:** Bing Cao, Chenbo Sun, Rui Bi, Zebing Liu, Yijun Jia, Wenli Cui, Menghong Sun, Baohua Yu, Xiaoqiu Li, Xiaoyan Zhou

**Affiliations:** 1 Department of Pathology, Fudan University Shanghai Cancer Center, Shanghai, China; 2 Department of Oncology, Shanghai Medical College, Fudan University, Shanghai, China; 3 Institute of Pathology, Fudan University, Shanghai, China; 4 Fudan University Medical Library, Shanghai, China; 5 Department of Pathology, Renji Hospital, School of Medicine, Shanghai Jiao Tong University, Shanghai, China; 6 Department of Pathology, The First Affiliated Hospital of Xinjiang Medical University, Urumqi, Xinjiang Uygur Autonomous Region China

**Keywords:** Diffuse large B-cell lymphoma, Targeted sequencing, Next-generation sequencing, Mutation, Genetic subtype

## Abstract

**Background:**

Diffuse large B-cell lymphoma (DLBCL), an aggressive and heterogenic malignant entity, is still a challenging clinical problem, since around one-third of patients are not cured with primary treatment. Next-generation sequencing (NGS) technologies have revealed common genetic mutations in DLBCL. We devised an NGS multi-gene panel to discover genetic features of Chinese nodal DLBCL patients and provide reference information for panel-based NGS detection in clinical laboratories.

**Methods:**

A panel of 116 DLBCL genes was designed based on the literature and related databases. We analyzed 96 Chinese nodal DLBCL biopsy specimens through targeted sequencing.

**Results:**

The most frequently mutated genes were *KMT2D* (30%), *PIM1* (26%), *SOCS1* (24%), *MYD88* (21%), *BTG1* (20%), *HIST1H1E* (18%), *CD79B* (18%), *SPEN* (17%), and *KMT2C* (16%). *SPEN* (17%) and *DDX3X* (6%) mutations were highly prevalent in our study than in Western studies. Thirty-three patients (34%) were assigned as genetic classification by the LymphGen algorithm, including 12 cases MCD, five BN2, seven EZB, seven ST2, and two EZB/ST2 complex. *MYD88* L265P mutation, *TP53* and *BCL2* pathogenic mutations were unfavorable prognostic biomarkers in DLBCL.

**Conclusions:**

This study presents the mutation landscape in Chinese nodal DLBCL, highlights the genetic heterogeneity of DLBCL and shows the role of panel-based NGS to prediction of prognosis and potential molecular targeted therapy in DLBCL. More precise genetic classification needs further investigations.

**Supplementary Information:**

The online version contains supplementary material available at 10.1186/s12920-024-01866-y.

## Background

Diffuse large B-cell lymphoma (DLBCL) is the commonest type of adult lymphoma worldwide, comprising 30%–40% of non-Hodgkin lymphoma (NHL) [1]. In China, DLBCL accounts for 37.9% of cases of NHL [2]. Although approximately 70% of DLBCL cases are curable using frontline immunochemotherapy with R-CHOP (rituximab, cyclophosphamide, doxorubicin, vincristine, and prednisone) [3, 4], one-third of patients are refractory to primary treatment or relapse after therapy [5]. One explanation for this incomplete therapeutic success is the heterogeneity of this disease. Gene expression profiling (GEP) has uncovered two major ‘‘cell-of-origin’’ (COO) subtypes arising from B cells in different phases of differentiation, namely germinal center B-cell-like (GCB) DLBCL and activated B-cell-like (ABC) DLBCL, which have distinct biology and survival rates [6]. As GEP is poorly available in routine clinical practice, the COO on basis of immunohistochemistry was founded by the Hans algorithm [7]. Immunohistochemical biomarkers (CD10, BCL6, and MUM1) are used to define the GCB and non-GCB subgroups of DLBCL and forecast survival similar to the GEP [7].

In recent years, next-generation sequencing (NGS) has rendered new insights on the genomic features of DLBCL by discovering novel mutation targets via whole genome sequencing (WGS) and whole exome sequencing (WES) [8–17]. Based on the results of in-depth genomic analyses, new classifications for DLBCL have been proposed. These classifications divide DLBCL into distinct subtypes with discrete genetic signatures [14–17]. Staudt and colleagues [14, 15] developed the LymphGen algorithm, and identified six genetic subtypes of DLBCL, including MCD (*MYD88* L265P and *CD79B* co-mutated), BN2 (*BCL6* fusions and *NOTCH2* mutations), N1 (*NOTCH1* mutations), EZB (*EZH2* mutations and *BCL2* translocations), ST2 (*SGK1* and *TET2* mutations), and A53 (*TP53* mutations and deletions).

As WGS or WES is still comparatively expensive and has lower sequencing depth, targeted sequencing is commonly used in currently ongoing studies. Furthermore, clinical laboratories have been launching disease-targeted sequencing tests based on multi-gene panels [18]. In the current study, we designed a panel of 116 genes for DLBCL, and sequenced 96 Chinese nodal DLBCL patients via targeted sequencing. Our purpose was to observe the mutation landscape of Chinese nodal DLBCL and their relationship with clinicopathological characteristics, and to provide certain reference information for panel-based NGS detection in clinical laboratories.

## Materials and methods

### Patients

A total of 277 frozen lymph node samples with DLBCL were collected from Fudan University Shanghai Cancer Center between November 2005 and August 2014. Clinical information was collected through a review of medical charts. The inclusion criteria included newly diagnosed with DLBCL, previously untreated, treated with at least four cycles of R-CHOP or CHOP regimen in the first-line therapy, and with available DNA of adequate quality extracted from frozen tumor samples. Patients with human immunodeficiency virus infection, primary mediastinal large B-cell lymphoma and incomplete clinical information were precluded from this study. The diagnosis of DLBCL was established according to the 2008 World Health Organization (WHO) classification [1]. Finally, 96 patients were enrolled for analysis. This study was approved by the Fudan University Shanghai Cancer Center Institutional Review Board, and was performed in accordance with the Declaration of Helsinki.

### Multi-gene panel design

A panel was devised to capture 116 DLBCL genes (Table S1), based on the literature [8–12, 19–29], the Catalogue of Somatic Mutations in Cancer (COSMIC) database, and the FoundationOne Heme (Foundation Medicine, Cambridge, MA, USA). Probes for all coding exons of the 116 genes were designed via the SureDesign tool (Agilent Technologies, Santa Clara, CA, USA).

### Illumina-based targeted sequencing

Genomic DNA from frozen tumor tissue was extracted according to the standard protocols of the QIAamp DNA Mini Kit (Qiagen, Hilden, Germany). DNA quality was examined by spectrophotometer (Nanodrop, Thermo Fisher Scientific Inc.) and agarose gel electrophoresis. The qualified genomic DNA was randomly fragmented, ligated with adapters, purified, and amplified by ligation-mediated PCR. Hybrid capture was conducted through the SureSelect Target Enrichment System (Agilent Technologies, Santa Clara, CA, USA). The resulting DNA libraries were then loaded on Illumina HiSeq 2500 platform (Illumina, San Diego, CA, USA) for sequencing. The mean reads mapping rate was 99.8%. The mean coverage sequencing depth on the official target reached 750.8X.

### Bioinformatic analysis of DNA variants

Sequence reads from the Illumina HiSeq instrument were mapped to the reference human genome (hg19) via Burrows-Wheeler Aligner software with the default parameters. Picard was used to mark duplicates and followed by Genome Analysis Toolkit (GATK) to raise alignment accuracy. Single nucleotide variants (SNVs) and short insertion/deletions (Indels) were detected with GATK. The called variants were further recalibrated and filtered to output reliable variant calls, and then annotated with ANNOVAR. Variants were manually reviewed via the Integrative Genomics Viewer (IGV) and verified by Sanger sequencing.

### Sanger sequencing

Sanger sequencing was conducted to validate some of the identified variants gained from the targeted sequencing. PCR primers were either sourced from published studies [30–35] or designed with Primer-BLAST. PCR products were purified and sequenced on an ABI 3130 sequencer (Applied Biosystems, Foster City, CA, USA).

### Statistical analysis

Statistical analysis was performed with the SPSS 22.0 software package (SPSS, Chicago, IL, USA). Categorical variables were compared through the chi-square test or Fisher's exact test when applicable. Progression-free survival (PFS) was calculated from the date of diagnosis to the date of first disease progression, relapse, death resulting from any cause, or last follow-up. Overall survival (OS) was measured from the time of initial diagnosis to the time of death from any cause or last follow-up. The PFS and OS rates were estimated by the Kaplan–Meier method. Univariate analysis was assessed by the log-rank test. Multivariate analysis was carried out by means of Cox proportional hazards model. *P* values were adjusted for multiple testing using the Benjamini–Hochberg method. *P* < 0.05 or false discovery rate (FDR) < 0.05 was considered statistically significant.

## Results

### Patient characteristics

In total, 96 DLBCL patients were enrolled. Seventy-six patients accepted R-CHOP regimen and 20 patients received CHOP therapy. The median age was 55 years. The Ann Arbor stage classification was as follows: stage I-II in 57 (59.4%) patients, and stage III-IV in 39 (40.6%) patients. Eighty-eight patients (91.7%) were International Prognostic Index (IPI) 0–2, and 8 cases were IPI 3–5. The median follow-up time was 35.8 months (range: 3.5–122.7 months). In terms of the Hans algorithm, 36 cases (37.5%) were classified as GCB, while 60 cases (62.5%) were assigned as non-GCB. After frontline therapy, the objective response rate (ORR) was 84.4%, with complete remission (CR) rate of 69.8% (67/96) and partial remission (PR) rate of 14.6% (14/96). The 5-year PFS was 62.1%, and the 5-year OS was 70%.

### Gene mutational status

**Fig. 1:**
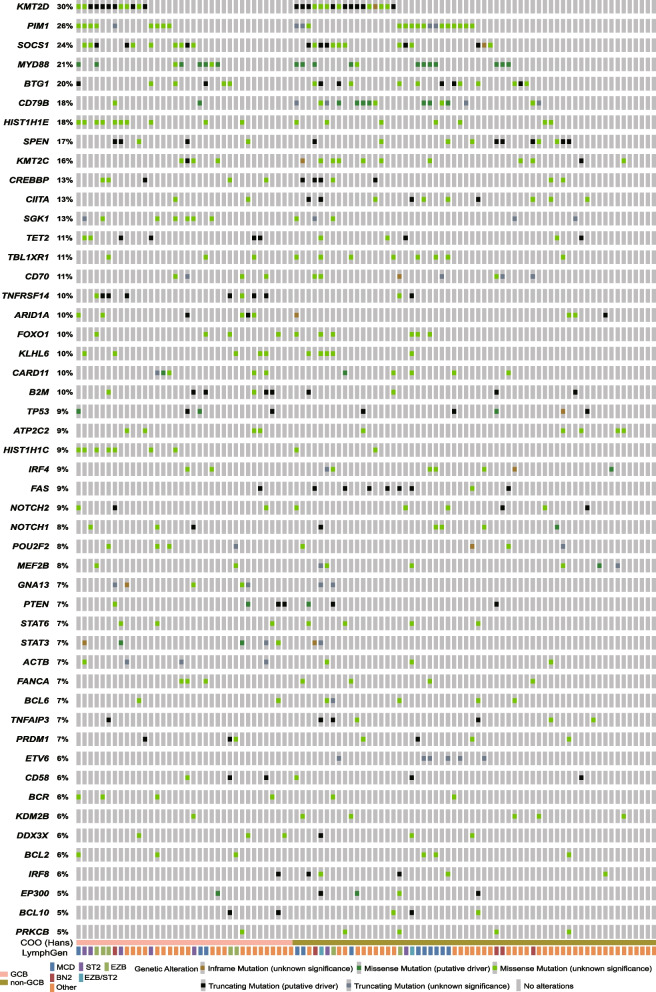
OncoPrint of non-silent mutations for the top genes in 96 Chinese DLBCL patients. Genes affected by non-silent mutations in at least 5 DLBCL samples (5% of cases) are listed. These mutations were annotated by OncoKB in cBioPortal on February 3, 2022. Each row represents a gene, and each column represents a DLBCL sample. Orange squares: inframe mutation (unknown significance); dark green squares: missense mutation (putative driver); light green squares: missense mutation (unknown significance); black squares: truncating mutation (putative driver); blue gray squares: truncating mutation (unknown significance); gray bars: no alterations. Nonsense, frameshift and splice site mutations are referred to as truncating mutations in the figure

Sequence data were filtered using the database of dbSNP142 and 1000Genome to remove germline variants with more than 0.1% population frequency reported previously. Then, variants in exons and splice sites except synonymous SNVs were kept. Mutation patterns included insertion, deletion, splice site, nonsense and missense mutation. All the 96 patients had at least one mutation of the 116 genes. A total of 899 non-silent somatic mutations were discovered, including 677 missense, 26 insertion, 67 deletion, 73 nonsense, and 56 splice site mutations. The data of genes with mutation frequency ≥ 5% were analyzed by cBioPortal for an overview of mutations in these genes affecting DLBCL samples (Fig. 1) [36]. These mutations were concurrently annotated by OncoKB in cBioPortal (Fig. 1).

Selected samples with identified mutations in *BTK, CARD11, CD79A, CD79B, EZH2, ETV6, FBXW7, MEF2B, MYD88, NOTCH1, NOTCH2, SPEN* and *TNFAIP3* were sent for Sanger sequencing for verification. All results of Sanger sequencing were consistent with the targeted sequencing.

Among the 116 genes, no somatic mutations were found in 11 genes. They are *AHR*, *AKT1*, *CCND2*, *CDKN2B*, *ID3*, *IDH1*, *KRAS*, *MAP3K7*, *RHOA*, *STAT5B*, and *TRAF3*. The top five genes with more genetic variants were *SOCS1* (86 SNVs), *PIM1* (68 SNVs), *KMT2D* (36 SNVs), *BTG1* (33 SNVs), and *SGK1* (33 SNVs).

The gene mutation frequencies were shown in Table S2. As for mutation frequency, the top five genes were *KMT2D* (30%), *PIM1* (26%), *SOCS1* (24%), *MYD88* (21%), and *BTG1* (20%). There were 49 genes whose mutation frequency was ≥ 5%, and the mutation frequency of 21 genes was ≥ 10% (Fig. 1).

By comparing the mutation frequency between our study and published papers, we found that *SPEN* mutation frequency in this study (17%) was higher than that in Western research (1–6%), and the mutation frequency of *DDX3X*, which was not previously described as mutated in Western DLBCL cases, was 6% in our study (Table S2).

### *SPEN* and *DDX3X* variants

**Fig. 2:**
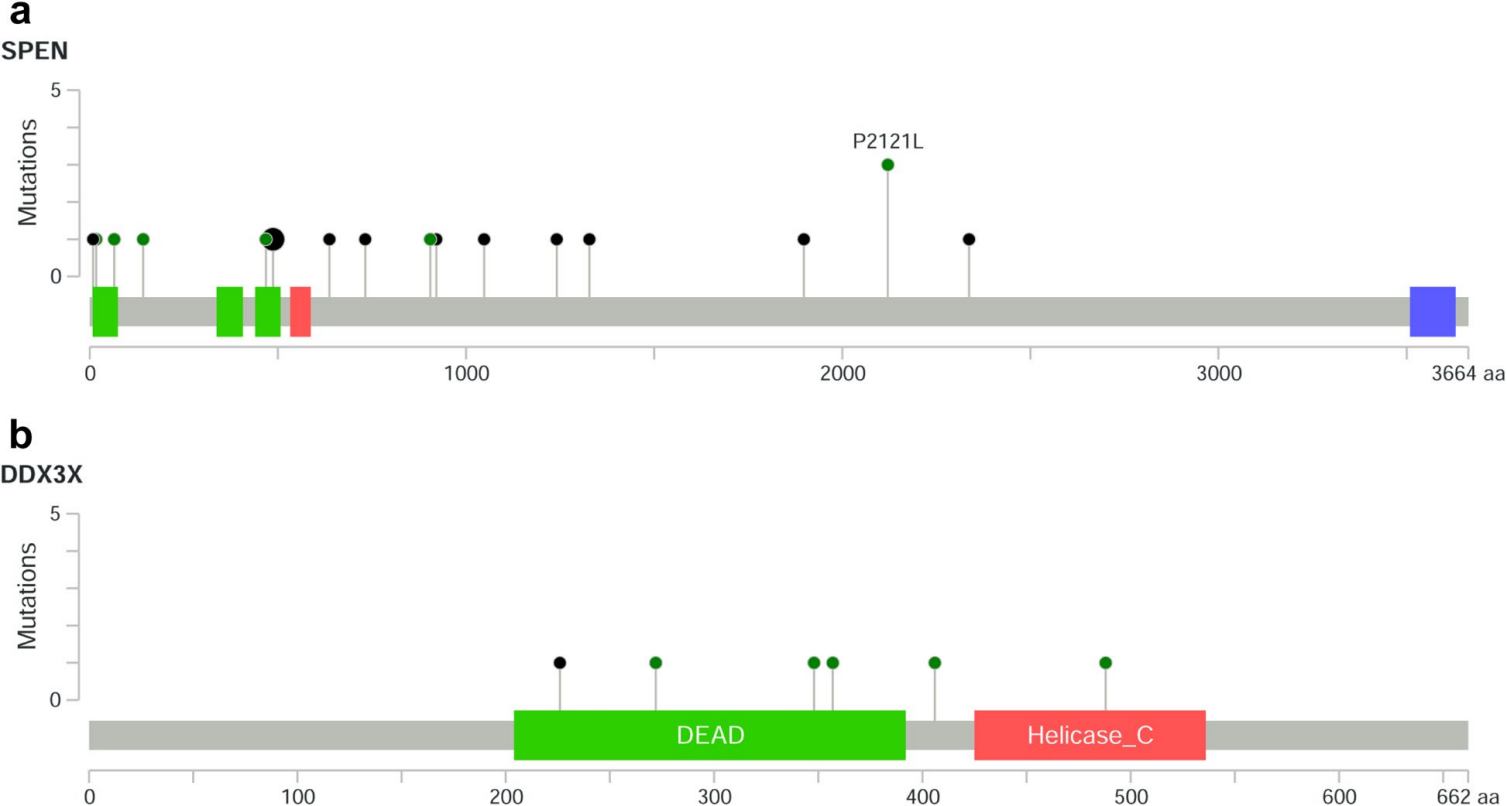
Schematic diagrams summarizing the mutations identified in *SPEN* (**a**) and *DDX3X* (**b**). The schematic diagrams were performed with the aid of MutationMapper in cBioPortal. Green circles: missense mutation; black circles: truncating mutation. Nonsense, frameshift and splice site mutations are referred to as truncating mutations in the figure

Eighteen mutations in *SPEN* were identified in 16 DLBCL patients, and only four of them were present in the COSMIC database (v92). Among the 18 *SPEN* SNVs, 8 (44%) were nonsense mutations, 2 (11%) were deletions, and 8 (44%) were missense mutations (Table S4, Fig. 2a). Twelve of the 18 (67%) mutations were located in exon 11 of *SPEN*, two (11%) in exon 1, two (11%) in exon 7, one (6%) in exon 2, and one (6%) in exon 3.

Six mutations in *DDX3X* were found in 6 DLBCL cases, including 5 missense mutations and 1 splice site mutation (Table S3, Fig. 2b). Four of these mutations were present in the COSMIC database (v92). Four of the 6 SNVs clustered in the ATP-binding helicase domain (residues 211–403), one mutation was located close to the ATP-binding helicase domain, and one mutation was located in the C-terminal helicase domain (residues 414–575).

### Genetic subtypes of DLBCL

**Fig. 3:**
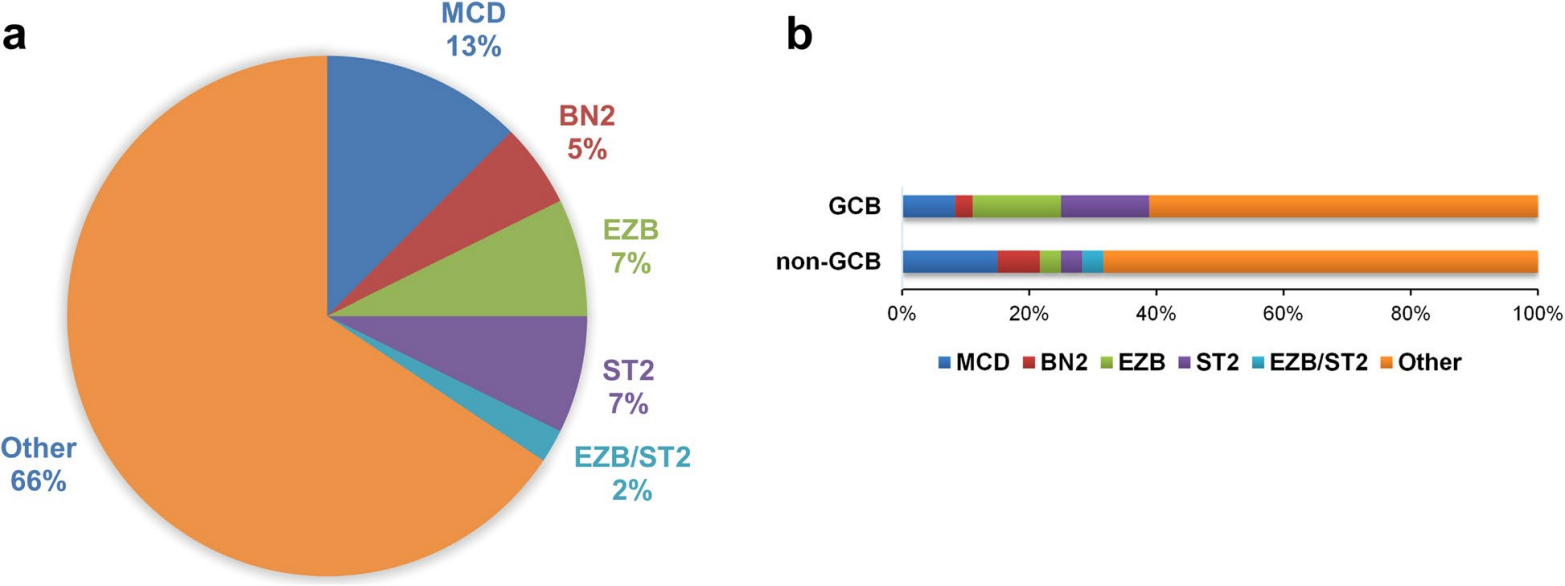
Genetic subtypes of DLBCL. **a** Prevalence of genetic subtypes of DLBCL classified by the LymphGen tool. **b** Prevalence of genetic subtypes in COO (Hans) subgroups

The LymphGen classification tool [15] accommodates mutation-only data from exome or targeted sequencing, but if lacking copy number variant (CNV) data, the A53 subtype cannot be identified. We used the LymphGen tool (https://llmpp.nih.gov/lymphgen/index.php) to analyze our cohort. Twelve cases were assigned as MCD, five as BN2, seven as EZB, seven as ST2, and two as EZB/ST2 complex. Sixty-three cases (66%) remained unclassified (Fig. 3a). Each of the COO (Hans) subtypes involved several genetic subgroups, with GCB cases enriched for EZB and ST2, and non-GCB cases enriched for MCD and BN2 (Fig. 3b).

There was no overlay between cases with *NOTCH1* and *NOTCH2* mutations. Only one patient had both *NOTCH1* and *SPEN* mutations. Mutual exclusivity analysis showed that *NOTCH1* and *NOTCH2* (FDR = 0.706), and *NOTCH1* and *SPEN* (FDR = 0.706) tended to occur in a mutually exclusive way. *NOTCH2* and *SPEN* (FDR = 0.706) tended to occur in a co-occurrence way. Twelve mutations in *TP53* were identified in 9 cases, containing 2 insertion, 1 deletion, 5 missense, and 4 splice site mutations.

### Pathway analysis

Seventy-four genes in the panel were grouped into specific pathways (Table 1): (i) B-cell receptor (BCR) signaling; (ii) Toll-like receptor (TLR) signaling; (iii)nuclear factor-κB (NF-κB) signaling; (iv) NOTCH signaling; (v) phosphatidylinositol 3-kinase (PI3K)/AKT signaling; (vi) Janus kinase/signal transducers and activators of transcription (JAK/STAT) signaling; (vii) mitogen-activated protein kinase (MAPK) signaling; (viii) sphingosine 1-phosphate receptor 2 (S1P2) signaling; (ix) epigenetic regulation; and (x) immunity.

**Table 1:** Mutation frequencies of genes in pathways

Pathway	Number of non-silent somatic mutations	Mutation frequency (%, *n* = 96)	Mutation frequency in GCB subtype (%, *n* = 36)	Mutation frequency in non-GCB subtype (%, *n* = 60)	*P*	FDR
**BCR signaling**						
*CD79A*	2	2	3	2	1.000	1.000
*CD79B*	23	18	6	25	0.016	0.536
*BTK*	3	3	6	2	0.650	1.000
*CARD11*	12	10	14	8	0.605	1.000
*MALT1*	5	2	3	2	1.000	1.000
*BCL10*	5	5	6	5	1.000	1.000
*PRKCB*	6	5	3	7	0.722	1.000
*TCF3*	4	4	6	3	1.000	1.000
*ID3*	0	0	0	0	NA	NA
**TLR signaling**						
*MYD88*	20	21	22	20	0.795	1.000
**NF-κB signaling**						
*TNFAIP3*	7	7	3	10	0.362	1.000
*TRAF2*	2	1	0	2	1.000	1.000
*TRAF5*	2	2	0	3	0.526	1.000
*MAP3K7*	0	0	0	0	NA	NA
*IKBKB*	1	1	0	2	1.000	1.000
*TNFRSF11A*	1	1	0	2	1.000	1.000
*TRAF3*	0	0	0	0	NA	NA
*BIRC3*	4	4	8	2	0.291	1.000
*MAP3K14*	1	1	3	0	0.375	1.000
*NFKBIA*	5	4	6	3	1.000	1.000
**NOTCH signaling**						
*NOTCH1*	8	8	8	8	1.000	1.000
*NOTCH2*	9	9	8	10	1.000	1.000
*SPEN*	18	17	14	18	0.572	1.000
*FBXW7*	5	4	3	5	1.000	1.000
**PI3K/AKT signaling**						
*PIK3CA*	2	2	3	2	1.000	1.000
*PIK3CD*	1	1	3	0	0.375	1.000
*PIK3CG*	4	4	3	5	1.000	1.000
*PIK3R1*	2	2	3	2	1.000	1.000
*PIK3R2*	2	2	0	3	0.526	1.000
*AKT1*	0	0	0	0	NA	NA
*AKT2*	1	1	0	2	1.000	1.000
*AKT3*	3	3	3	3	1.000	1.000
*PTEN*	10	7	11	5	0.478	1.000
*MTOR*	4	4	6	3	1.000	1.000
*SGK1*	33	13	19	8	0.202	1.000
**JAK/STAT signaling**						
*JAK1*	1	1	0	2	1.000	1.000
*JAK2*	4	4	3	5	1.000	1.000
*JAK3*	2	2	6	0	0.138	1.000
*STAT3*	10	7	14	3	0.128	1.000
*STAT5A*	2	1	0	2	1.000	1.000
*STAT5B*	0	0	0	0	NA	NA
*STAT6*	8	7	8	7	1.000	1.000
*SOCS1*	86	24	28	22	0.497	1.000
*PTPN1*	1	1	3	0	0.375	1.000
*MPL*	1	1	3	0	0.375	1.000
**MAPK signaling**						
*NRAS*	1	1	3	0	0.375	1.000
*KRAS*	0	0	0	0	NA	NA
*BRAF*	4	4	3	5	1.000	1.000
**S1P2 signaling**						
*GNA13*	9	7	14	3	0.128	1.000
*S1PR2*	4	4	3	5	1.000	1.000
**Epigenetic regulation**						
*ARID1A*	11	10	17	7	0.227	1.000
*CREBBP*	13	13	14	12	1.000	1.000
*EP300*	6	5	3	7	0.722	1.000
*DNMT3A*	2	2	0	3	0.526	1.000
*EZH2*	5	4	8	2	0.291	1.000
*HDAC1*	2	2	6	0	0.138	1.000
*HDAC4*	4	3	3	3	1.000	1.000
*HDAC7*	5	3	6	2	0.650	1.000
*HIST1H1C*	12	9	19	3	0.024	0.536
*HIST1H1E*	27	18	28	12	0.045	0.7538
*IDH1*	0	0	0	0	NA	NA
*IDH2*	1	1	0	2	1.000	1.000
*KDM2B*	7	6	3	8	0.514	1.000
*KMT2C*	16	16	11	18	0.345	1.000
*KMT2D*	36	30	33	28	0.605	1.000
*MEF2B*	8	8	6	10	0.703	1.000
*MEF2C*	1	1	0	2	1.000	1.000
*SETD2*	3	3	6	2	0.650	1.000
*TET2*	16	11	17	8	0.363	1.000
**Immunity**						
*B2M*	11	10	17	7	0.227	1.000
*CD58*	7	6	8	5	0.828	1.000
*CD70*	14	11	11	12	1.000	1.000
*CIITA*	14	13	6	17	0.202	1.000
*TNFRSF14*	11	10	22	3	0.010	0.536

*Abbreviation*: *GCB* germinal center B-cell-like, *FDR* false discovery rate, *NA* not available

Within the 96 specimens, 91 (95%) had at least one mutation in 74 pathway genes, including 34/36 in GCB (94%), and 57/60 in non-GCB (95%) (Table S4). On the whole, 570 somatic mutations were detected in 74 genes, including 412 missense, 17 insertion, 55 deletion, 58 nonsense, and 28 splice site mutations. There was no significant difference in the mutation frequencies of the 74 genes between the GCB and non-GCB subgroups (Table 1).

In the eight signaling pathways, the most frequently concurred pathways were PI3K/AKT and JAK/STAT signaling (18 concurrence out of 50 cases, 36%), and BCR and JAK/STAT signaling (17 concurrence out of 56 cases, 30%). TLR and MAPK signaling, and TLR and S1P2 signaling had none concurrence.

### Potential biomarkers for targeted therapy

Our panel included 14 genes (*CD79A*, *CD79B*, *MYD88*, *EZH2*, *CARD11*, *CREBBP*, *EP300*, *TNFAIP3*, *JAK3*, *SOCS1*, *STAT6*, *NOTCH1*, *NOTCH2*, and *SPEN*) as therapeutic targets, and mutations of these genes could assist to stratify patients according to therapeutic options (Table S5) [28, 29, 37, 38]. Seventy-two cases (75%) had mutations in these genes, indicating that these patients might be candidates for corresponding clinical drug trials.

In our cohort, for *CD79A*, two missense mutations (Y188S and Y188D) in the ITAM domain were found in two cases. For *CD79B*, 17 patients had 23 variations, mainly situated in the ITAM domain. Eight missense mutations affected the tyrosine at position 196 (Y196). Twenty missense mutations in *MYD88*, mostly located in the TIR domain, were found in 20 patients. The most common variation was L265P (12/20; 60%), followed by S243N (4/20; 20%). For *CARD11*, 12 mutations were detected in 10 cases, mainly situated in the coiled-coil domain. Seven patients had mutations in *TNFAIP3*. For *MEF2B*, eight patients had eight mutations. In our study, five patients (5%) had both *CD79B* and *MYD88* mutations, and 51 patients (53%) had mutations in these six genes.

### Clinicopathological relevance

In this study, insertion, deletion, nonsense, and splice site mutations were considered to be pathogenic. To evaluate the pathogenicity of the missense mutations, the predictive software Polymorphism Phenotyping v2 (Polyphen-2) was used. These variations predicted as probably damaging or possibly damaging through Polyphen-2 were considered pathogenic. The pathogenic mutation frequencies of 41 genes were ≥ 5% (Table 2). No significant correlations between the 41 gene mutations and age, stage, or IPI were found. There was no correlation between genetic mutations and treatment response to frontline therapy in our study.

**Table 2:** Pathogenic mutation frequencies of 41 genes by subtype

Gene	GCB		non-GCB		*P*	FDR
	Number of cases of pathogenic mutation	Pathogenic mutation frequency (%, *n* = 36)	Number of cases of pathogenic mutation	Pathogenic mutation frequency (%, *n* = 60)		
*KMT2D*	8	22	15	25	0.758	1.000
*SOCS1*	8	22	10	17	0.500	0.996
*MYD88*	6	17	12	20	0.685	0.996
*MYD88 L265P*	4	11	8	13	1.000	1.000
*CD79B*	2	6	14	23	0.024	0.320
*PIM1*	4	11	10	17	0.455	0.996
*BTG1*	4	11	10	17	0.455	0.996
*SPEN*	3	8	8	13	0.679	0.996
*TBL1XR1*	3	8	8	13	0.679	0.996
*SGK1*	7	19	3	5	0.058	0.464
*HIST1H1E*	7	19	3	5	0.058	0.464
*CREBBP*	5	14	5	8	0.605	0.996
*TET2*	4	11	5	8	0.928	1.000
*KLHL6*	4	11	5	8	0.928	1.000
*TNFRSF14*	7	19	2	3	0.024	0.320
*B2M*	5	14	4	7	0.416	0.996
*FAS*	1	3	8	13	0.175	0.875
*TP53*	4	11	5	8	0.928	1.000
*KMT2C*	2	6	6	10	0.703	0.996
*CIITA*	0	0	8	13	NA	NA
*CARD11*	5	14	3	5	0.253	0.996
*IRF4*	1	3	7	12	0.253	0.996
*NOTCH2*	2	6	6	10	0.703	0.996
*POU2F2*	4	11	4	7	0.703	0.996
*HIST1H1C*	6	17	1	2	0.020	0.320
*MEF2B*	1	3	6	10	0.362	0.996
*NOTCH1*	3	8	4	7	1.000	1.000
*GNA13*	5	14	2	3	0.128	0.731
*STAT3*	5	14	2	3	0.128	0.731
*STAT6*	3	8	4	7	1.000	1.000
*ETV6*	0	0	6	10	NA	NA
*CD70*	1	3	5	8	0.514	0.996
*BCL6*	2	6	4	7	1.000	1.000
*ACTB*	4	11	2	3	0.276	0.996
*CD58*	3	8	3	5	0.828	1.000
*ARID1A*	3	8	2	3	0.553	0.996
*FOXO1*	3	8	2	3	0.553	0.996
*PRDM1*	3	8	2	3	0.553	0.996
*PTEN*	3	8	2	3	0.553	0.996
*BCL2*	2	6	3	5	1.000	1.000
*DDX3X*	2	6	3	5	1.000	1.000
*EP300*	1	3	4	7	0.722	0.996

*Abbreviation*: *GCB* germinal center B-cell-like, *FDR* false discovery rate, *NA* not available

**Fig. 4:**
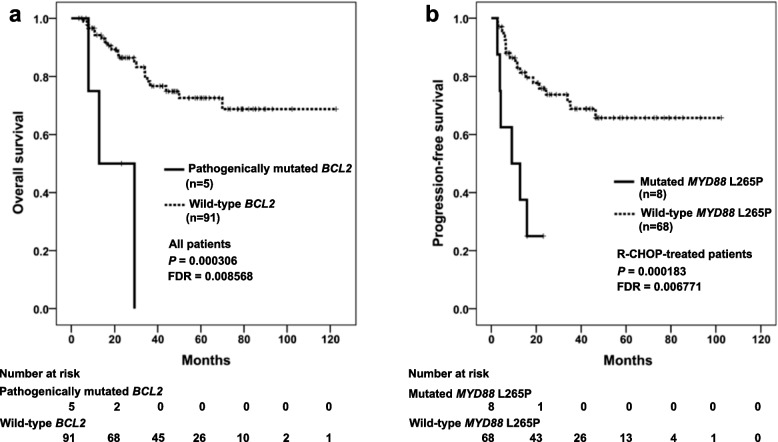
Kaplan–Meier curves for progression-free survival and overall survival of patients with DLBCL. **a** Survival analysis was performed on total patients according to *BCL2* mutation status. **b** Survival analysis was performed on patients treated with R-CHOP according to the presence or absence of *MYD88* L265P mutation

Univariate prognostic analysis was assessed for 41 genes in all 96 patients and 76 patients receiving the R-CHOP regimen separately (Table S6). Among all 96 patients, *BCL2* pathogenic mutations were significantly associated with worse OS (FDR = 0.008568, Fig. 4a). In the R-CHOP group, *MYD88* L265P–mutated patients had significantly lower PFS (FDR = 0.006771, Fig. 4b).

Important factors (*KMT2D, MYD88, MYD88* L265P, *TP53,* and *BCL2*) from the univariate analyses for PFS and OS (Table S6), and age, stage, IPI, LDH, and COO (Hans) subtypes were included in the multivariate Cox regression analysis. In addition, treatment regimen was added as a factor in the all patients cohort. Among all patients, multivariate analysis demonstrated that the *MYD88* L265P mutation (HR, 2.689; *P* = 0.017), *TP53* pathogenic mutations (HR, 3.129; *P* = 0.015), stage III-IV (HR, 3.882; *P* < 0.001), and non-GCB (HR, 2.283; *P* = 0.042) were independently associated with shorter PFS. *BCL2* pathogenic mutations (HR, 6.364; *P* = 0.007) were independently correlated with shorter OS. In the R-CHOP group, multivariate analysis revealed that the *MYD88* L265P mutation (HR, 4.321; *P* = 0.003) and stage III-IV (HR, 2.665; *P* = 0.021) were independently correlated with worse PFS, and *BCL2* pathogenic mutations (HR, 6.705; *P* = 0.016) were independently correlated with worse OS. Immunohistochemical data for BCL2 in 86 patients were analyzed. With the 50% cutoff, the expression of BCL2 was assessed for positivity or negativity. BCL2 was positive in 57% of the cases (*n* = 49), and negative in 43% of the cases (*n* = 37). IHC analysis revealed that BCL2 expression was not associated with PFS (*P* = 0.966) or OS (*P* = 0.736). There was no relationship between BCL2 expression and *BCL2* mutation (Spearman rho = 0.037, *P* = 0.734).

Among the thirty-three patients assigned to the LymphGen subgroup, the treatment response of two MCD patients to frontline therapy was progressive disease (PD), and the response of the other patients was CR or PR. Fisher's exact test showed no difference in treatment response within genetic subgroups. We assessed the survival of patients classified into LymphGen subgroups. For all patients or the R-CHOP-treated patients, although not statistically significant, the PFS of MCD patients was inferior to that of non-MCD patients (*P* = 0.059 and *P* = 0.060, separately). Within all patients, ST2 patients had favorable PFS and OS compared with MCD patients (*P* = 0.059 and *P* = 0.061, separately), and had favorable OS compared with BN2 patients (*P* = 0.062). Among R-CHOP–treated patients, ST2 patients had significantly better PFS than MCD patients (*P* = 0.048). ST2 patients had favorable OS compared with MCD (*P* = 0.063) and BN2 (*P* = 0.062) patients.

## Discussion

DLBCL remains a challenging clinical puzzle, as about one-third of patients not being cured by the R-CHOP regimen. The limitations to effective therapy are partly relative to the heterogeneity of this disease. In this study, we utilized a panel-based NGS strategy to identify the mutation landscape in 96 Chinese DLBCL patients.

The most recurrently mutated gene in our cohort was *KMT2D* (30%), in accordance with other NGS studies [8–10]. *KMT2D*, also known as *MLL2*, encodes a conserved histone methyltransferase that regulates gene transcription via methylating the lysine-4 position of histone H3 (H3K4) [39]. In our study, of 36 *KMT2D* SNVs, 25% were nonsense mutations, 25% were deletions, 8% were splice site mutations, and 42% were missense mutations. We did not find apparent hotspots. The truncated proteins generated from nonsense, deletion and splice site mutations lack the C-terminal SET domain needed for enzymatic activation, indicating that *KMT2D* is a tumor suppressor [8–10]. Recently, Zhang et al. [40] reported that *KMT2D* missense mutations affecting the C-terminal SET domain impaired KMT2D methyltransferase activity, resulting in reduction of H3K4 methylation. Moreover, Ortega-Molina et al. [41] showed that *KMT2D* mutations may promote lymphoma development by disturbing the expression of tumor repressor genes that regulate B cell–activating pathways. Clinically, we found that *KMT2D* pathogenic mutations were not related to survival in DLBCL patients, which was consistent with the findings of studies by Ortega-Molina et al. [41] and Dubois et al. [42]. Additional studies with more patients are needed to further analyze the clinical significance of *KMT2D* mutations.

*SPEN* was affected by 18 mutations in 17% of the sequenced patients, whose mutation frequency was higher than that in Western studies (1–6%). To our knowledge, this is the first report describing *SPEN* as a frequently mutated target in DLBCL. *SPEN* (aliases: *MINT* and *SHARP*), encodes a hormone inducible transcriptional repressor. This protein is characterized by four RNA recognition motifs at the N-terminus and a highly conservative SPOC domain at the C-terminus. It also contains several nuclear localization sequences, a region interacting with MSX2, and a region interacting with RBPJ [43]. SPEN has been shown to compete with the NOTCH endocellular domain for attaching to RBPJ and to repress the transactivation activity of NOTCH signaling [44, 45]. In addition, SPEN is engaged in transcriptional suppression in several systems other than the NOTCH signaling pathway, such as the MSX2 and Ras/MAPK signaling pathways [46, 47]. Recent studies have shown that *SPEN* is mutated in 5% of splenic marginal zone lymphoma cases [48], and *SPEN* functions as a tumor repressor and candidate biomarker of tamoxifen responsiveness in ERα-positive breast cancers [49].

*DDX3X* (aliases: *DBX*, *DDX3* and *CAP-Rf*), an ATP-dependent RNA helicase gene, lies on chromosome X with functions in RNA transcription, RNA splicing, mRNA transport, translation, and cell cycle modulation [50]. Owing to its diverse role in RNA metabolism, *DDX3X* has received growing interest of its role in cancer. Predominantly missense mutations of *DDX3X* were identified in our DLBCL samples, which was similar to the mutational spectrum in medulloblastoma [51–53]. Whereas, the mutant spectrum of *DDX3X* in DLBCL was dissimilar to that in natural killer/T-cell lymphoma and chronic lymphocytic leukemia, in which mostly truncating mutations (nonsense, frameshift or splice site) have been identified [54, 55]. *DDX3X* has been reported to be both a tumorigenesis gene and a tumor repressor [56]. The function of DDX3X in DLBCL remains to be determined.

Staudt and colleagues [15] applied the LymphGen classification tool to DLBCL samples from an NCI patient cohort (*n* = 574), and the tool classified 63% of the cases. When we classified our cohort using the LymphGen tool [15], 33 cases (34%) were assigned as genetic classification, including 12 cases MCD, five BN2, seven EZB, seven ST2, and two EZB/ST2 complex. Sixty-three cases (66%) remained unclassified. This may be on account of the lack of CNV data, and information regarding *BCL2* translocations and *BCL6* translocations. These data need to be supplemented in the future. The distribution of genetic subtypes in COO (Hans) subgroups in our cohort was consistent with the distribution of subtypes in COO (GEP) subgroups in the study by Staudt and colleagues [15]. Despite functional similarities between NOTCH1 and NOTCH2, Staudt and colleagues speculated that the pathogenesis of N1 subtype was distinct from that of BN2 subtype based on the differences in genetics, phenotype, and clinic [14]. Although none were assigned as N1 subtype in our cohort, there was no overlap in specimens with *NOTCH1* or *NOTCH2* mutations.

Previous studies have shown the therapeutic potential of sotrastaurin, a selective protein kinase C inhibitor, in patients with *CD79A/B* mutant DLBCL, whereas *CARD11* mutations rendered insensitive [57]. Furthermore, *CARD11* mutation has been shown to confer DLBCL cell resistance to lenalidomide, an orally administered immunomodulatory drug [58]. A phase I/II clinical trial has revealed that DLBCL patients having both *CD79B* and *MYD88* mutations were more responsive to the BTK inhibitor ibrutinib, whereas *CARD11* mutations and TNFAIP3 inactivation (*TNFAIP3* nonsense or frameshift mutation, *TNFAIP3* double deletion, or low *TNFAIP3* mRNA expression) were associated with inferior responses to ibrutinib [59]. In a phase II study, *MEF2B*-mutant patients with relapsed/refractory DLBCL had a higher response rate to panobinostat, an HDAC inhibitor, than those without *MEF2B* mutations [60].

Zhang et al. [11] compared the gene mutations from four Western DLBCL NGS studies. They discovered fairly modest overlaps, and even genes overlapped between different studies often varied, suggesting that DLBCL has considerable genetic heterogeneity [11]. Moreover, de Miranda et al. [13] identified 11 novel genes (*TMSB4X*, *DCDC5*, *IGLL5*, *SLITRK3*, *CDKN2A*, *GPR37*, *LYN*, *OR10A2*, *PRDM15*, *TDRD6*, and *DDX3X*) with recurrent mutations in Chinese DLBCL, with the addition of the findings of our study, underscoring the influence of ethnic diversity on somatic alterations.

Recently, a simplified 20-gene algorithm for genetic subtyping was established using targeted sequencing and FISH analysis [61]. DLBCL patients were divided into 6 genetic subtypes (MCD-like, BN2-like, N1-like, EZB-like, *TP53*^mut^, and not otherwise specified). This classification was based on mutation data of 18 genes (*BTG1*, *CD70*, *CD79B*, *CREBBP*, *DTX1*, *EP300*, *EZH2*, *MPEG1*, *MTOR*, *MYD88*, *NOTCH1*, *NOTCH2*, *PIM1*, *STAT6*, *TBL1XR1*, *TNFAIP3*, *TNFRSF14*, and *TP53*) and re-arrangement data of 2 genes (*BCL2* and *BCL6*). The researchers found that R-CHOP combined with targeted agents (R-CHOP-X) based on the 6 genetic subtypes improved the CR rate, PFS and OS in patients with DLBCL in the GUIDANCE-01 trial.

There are several limitations to this study. First, the number of samples was small, and 91.7% of patients were in the low or low-intermediate risk group according to IPI. These factors led to the sample selection bias; therefore, further studies with a larger number of patients are needed. Second, we were unable to classify the A53 subtype (*TP53* mutations and deletions) due to the lack of CNV data.

## Conclusions

In conclusion, our work identified the genetic features of Chinese DLBCL patients. The most frequently mutated genes were *KMT2D* (30%), *PIM1* (26%), *SOCS1* (24%), *MYD88* (21%), *BTG1* (20%), *HIST1H1E* (18%), *CD79B* (18%), *SPEN* (17%), and *KMT2C* (16%). *SPEN* (17%) and *DDX3X* (6%) mutations were highly prevalent in our study than in Western studies. *MYD88* L265P mutation, *TP53* and *BCL2* pathogenic mutations were unfavorable prognostic biomarkers in DLBCL. Thirty-three cases (34%) were assigned as genetic classification by the LymphGen algorithm. Additional studies with more specimens will be demanded to further analyze the prognostic significance of each genetic subtype. Seventy-two patients (75%) had mutations in potentially targeted therapeutic genes, and 51 cases (53%) had mutations that potentially predicted drug response or resistance. To achieve the aim of precise treatment in DLBCL, a consensus gene panel incorporating somatic mutations with proven diagnostic, prognostic and/or therapeutical relevancy must be designed.

### Supplementary Information

**Supplementary Material 1.**

## Data Availability

The raw sequence data during the current study have been deposited in the Genome Sequence Archive in National Genomics Data Center, China National Center for Bioinformation / Beijing Institute of Genomics, Chinese Academy of Sciences (GSA-Human: HRA005331) that are publicly accessible at https://ngdc.cncb.ac.cn/gsa-human.
