# Supplementary material for: Mutation landscape in Chinese nodal diffuse large B-cell lymphoma by targeted next generation sequencing and their relationship with clinicopathological characteristics
Source: BMC Med Genomics. 2024 Apr 13;17:84. doi: 10.1186/s12920-024-01866-y (PMC11015559; doi:10.1186/s12920-024-01866-y)
Supplement: Supplementary file 1 — Supplementary Material 1. [file 12920_2024_1866_MOESM1_ESM.pdf]

Table S1 List of genes included in the designed panel

Gene									
ACTB	*AHR*	*AKT1*	*AKT2*	*AKT3*	*ARID1A*	*ARNT*	*ATM*	*ATP2C2*	*B2M*
BCL10	*BCL2*	*BCL6*	*BCR*	*BIRC3*	*BRAF*	*BRCA2*	*BTG1*	*BTK*	*CARD11*
CCND2	*CCND3*	*CD44*	*CD58*	*CD70*	*CD79A*	*CD79B*	*CDKN2A*	*CDKN2B*	*CIITA*
COL4A2	*CREBBP*	*DDX3X*	*DNMT3A*	*EP300*	*ETV6*	*EZH2*	*FANCA*	*FAS*	*FBXW7*
FOXO1	*GNA13*	*GNAI2*	*HDAC1*	*HDAC4*	*HDAC7*	*HIST1H1C*	*HIST1H1E*	*HMGB1*	*ID3*
IDH1	*IDH2*	*IKBKB*	*IRF4*	*IRF8*	*JAK1*	*JAK2*	*JAK3*	*KDM2B*	*KIT*
KLHL6	*KMT2C*	*KMT2D*	*KRAS*	*MALT1*	*MAP3K14*	*MAP3K7*	*MEF2B*	*MEF2C*	*MPL*
MTOR	*MYC*	*MYD88*	*NFKBIA*	*NOTCH1*	*NOTCH2*	*NRAS*	*P4HA2*	*PAX5*	*PDGFRA*
PIK3CA	*PIK3CD*	*PIK3CG*	*PIK3R1*	*PIK3R2*	*PIM1*	*POU2F2*	*PRDM1*	*PRKCB*	*PTEN*
PTPN1	*RHOA*	*RHOH*	*S1PR2*	*SETD2*	*SGK1*	*SOCS1*	*SPEN*	*SPIB*	*STAT3*
STAT5A	*STAT5B*	*STAT6*	*TBC1D10C*	*TBL1XR1*	*TCF3*	*TET2*	*TMEM30A*	*TNFAIP3*	*TNFRSF11A*
TNFRSF14	*TP53*	*TRAF2*	*TRAF3*	*TRAF5*	*UNC5C*				

Table S2 Mutation frequencies comparison between our and Western studies on DLBCL

Gene	Our study	Morin et al.*(1)	Pasqualucci et al.*(2)	Lohr et al.*(3)	Zhang et al.*(4)	Mottok et al.(5)	Schif et al.(6)	Arcaini et al.(7)	Asmar et al.(8)	Quivoron et al.(9)	Trinh et al.(10)
KMT2D	30%	32%	23%	29%	—	—	—	—	—	—	—
PIM1	26%	17%	14%	31%	12%	—	—	—	—	—	—
SOCS1	24%	—	—	6%	3%	27%	16%	—	—	—	—
MYD88	21%	13%	8%	12%	15%	—	—	—	—	—	—
BTG1	20%	14%	—	16%	10%	—	—	—	—	—	—
HIST1H1E	18%	—	—	14%	7%	—	—	—	—	—	—
CD79B	18%	8%	12%	16%	8%	—	—	—	—	—	—
SPEN	17%	—	—	6%	—	—	—	1%	—	—	—
KMT2C	16%	—	—	4%	18%	—	—	—	—	—	—
CREBBP	13%	15%	18%	16%	4%	—	—	—	—	—	—
SGK1	13%	20%	—	10%	5%	—	—	—	—	—	—
CIITA	13%	5%	—	10%	—	—	—	—	—	—	—
TBL1XR1	11%	—	—	6%	—	—	—	—	—	—	—
TET2	11%	6%	—	0%	—	—	—	—	12%	6%	—
CD70	11%	7%	—	10%	—	—	—	—	—	—	—
ARID1A	10%	—	—	4%	5%	—	—	—	—	—	—
FOXO1	10%	5%	—	0%	—	—	—	—	—	—	9%
KLHL6	10%	10%	—	8%	—	—	—	—	—	—	—
TNFRSF14	10%	8%	—	22%	—	—	—	—	—	—	—
CARD11	10%	10%	10%	20%	11%	—	—	—	—	—	—
B2M	10%	8%	13%	10%	4%	—	—	—	—	—	—
ATP2C2	9%	—	—	2%	—	—	—	—	—	—	—
HIST1H1C	9%	9%	—	14%	8%	—	—	—	—	—	—
IRF4	9%	9%	—	2%	—	—	—	—	—	—	—
NOTCH2	9%	—	—	4%	7%	—	—	9%	—	—	—
FAS	9%	4%	—	0%	—	—	—	—	—	—	—
TP53	9%	12%	17%	24%	5%	—	—	—	—	—	—
MEF2B	8%	15%	8%	18%	3%	—	—	—	—	—	—
NOTCH1	8%	—	2%	8%	—	—	—	2%	—	—	—
POU2F2	8%	—	—	8%	5%	—	—	—	—	—	—
FANCA	7%	—	—	2%	—	—	—	—	—	—	—
GNA13	7%	15%	—	20%	7%	—	—	—	—	—	—
STAT3	7%	9%	—	10%	—	—	—	—	—	—	—
STAT6	7%	5%	—	4%	—	—	—	—	—	—	—
BCL6	7%	8%	—	8%	3%	—	—	—	—	—	—
PRDM1	7%	—	12%	0%	1%	—	—	—	—	—	—
TNFAIP3	7%	3%	15%	2%	10%	—	—	—	—	—	—
ACTB	7%	—	—	10%	—	—	—	—	—	—	—
PTEN	7%	—	—	2%	—	—	—	—	—	—	—
ETV6	6%	—	—	6%	—	—	—	—	—	—	—
BCL2	6%	28%	9%	22%	4%	—	—	—	—	—	—
BCR	6%	—	—	10%	—	—	—	—	—	—	—
CD58	6%	5%	6%	10%	—	—	—	—	—	—	—
KDM2B	6%	—	7%	2%	—	—	—	—	—	—	—
DDX3X	6%	—	—	0%	—	—	—	—	—	—	—
IRF8	6%	12%	—	6%	5%	—	—	—	—	—	—
BCL10	5%	4%	—	2%	—	—	—	—	—	—	—
EP300	5%	4%	5%	6%	—	—	—	—	—	—	—
PRKCB	5%	—	—	4%	—	—	—	—	—	—	—
BIRC3	4%	—	—	0%	—	—	—	—	—	—	—
BRCA2	4%	1%	—	4%	5%	—	—	—	—	—	—
EZH2	4%	22%	6%	14%	1%	—	—	—	—	—	—
FBXW7	4%	—	—	4%	5%	—	—	—	—	—	—
JAK2	4%	—	—	0%	—	—	—	—	—	—	—
MTOR	4%	—	—	0%	3%	—	—	—	—	—	—
MYC	4%	—	6%	8%	1%	—	—	—	—	—	—
NFKBIA	4%	—	—	8%	—	—	—	—	—	—	—
PAX5	4%	—	—	2%	—	—	—	—	—	—	—

PIK3CG	4%	—	—	2%	—	—	—	—	—	—	—
S1PR2	4%	2%	—	6%	—	—	—	—	—	—	—
BRAF	4%	—	—	4%	—	—	—	—	—	—	—
CDKN2A	4%	—	—	0%	—	—	—	—	—	—	—
TCF3	4%	—	—	2%	—	—	—	—	—	—	—
COL4A2	4%	6%	—	0%	—	—	—	—	—	—	—
BTK	3%	—	—	4%	—	—	—	—	—	—	—
HDAC4	3%	—	—	0%	—	—	—	—	—	—	—
HDAC7	3%	7%	—	2%	—	—	—	—	—	—	—
P4HA2	3%	—	—	0%	—	—	—	—	—	—	—
SETD2	3%	—	—	4%	5%	—	—	—	—	—	—
AKT3	3%	—	—	0%	—	—	—	—	—	—	—
ARNT	2%	—	—	0%	—	—	—	—	—	—	—
ATM	2%	—	—	0%	—	—	—	—	—	—	—
CCND3	2%	6%	6%	6%	—	—	—	—	—	—	—
CD79A	2%	—	—	0%	—	—	—	—	—	—	—
DNMT3A	2%	—	—	2%	—	—	—	—	—	—	—
GNAI2	2%	—	—	4%	—	—	—	—	—	—	—
HDAC1	2%	—	—	0%	—	—	—	—	—	—	—
JAK3	2%	—	—	0%	—	—	—	—	—	—	—
MALT1	2%	3%	—	0%	—	—	—	—	—	—	—
PDGFRA	2%	—	—	0%	4%	—	—	—	—	—	—
PIK3CA	2%	—	—	2%	—	—	—	—	—	—	—
PIK3R1	2%	—	—	2%	4%	—	—	—	—	—	—
PIK3R2	2%	—	—	2%	—	—	—	—	—	—	—
SPIB	2%	3%	—	0%	—	—	—	—	—	—	—
TMEM30A	2%	4%	6%	4%	5%	—	—	—	—	—	—
TRAF5	2%	—	—	0%	—	—	—	—	—	—	—
UNC5C	2%	—	—	10%	—	—	—	—	—	—	—
AKT2	1%	—	—	0%	—	—	—	—	—	—	—
CD44	1%	—	—	0%	—	—	—	—	—	—	—
HMGB1	1%	—	2%	0%	—	—	—	—	—	—	—
IDH2	1%	—	—	0%	—	—	—	—	—	—	—
IKBKB	1%	—	—	0%	—	—	—	—	—	—	—
JAK1	1%	—	—	0%	—	—	—	—	—	—	—
KIT	1%	—	—	0%	1%	—	—	—	—	—	—
MAP3K14	1%	—	—	0%	—	—	—	—	—	—	—
MEF2C	1%	—	—	4%	—	—	—	—	—	—	—
MPL	1%	—	—	0%	5%	—	—	—	—	—	—
NRAS	1%	—	—	0%	—	—	—	—	—	—	—
PIK3CD	1%	2%	—	2%	4%	—	—	—	—	—	—
PTPN1	1%	—	—	0%	—	—	—	—	—	—	—
RHOH	1%	—	—	0%	—	—	—	—	—	—	—
STAT5A	1%	—	—	0%	—	—	—	—	—	—	—
TBC1D10C	1%	—	—	0%	—	—	—	—	—	—	—
TNFRSF11	1%	—	—	0%	—	—	—	—	—	—	—
TRAF2	1%	—	—	0%	—	—	—	—	—	—	—
AHR	0%	—	—	0%	4%	—	—	—	—	—	—
AKT1	0%	—	—	0%	—	—	—	—	—	—	—
CCND2	0%	—	—	0%	—	—	—	—	—	—	—
CDKN2B	0%	—	—	0%	—	—	—	—	—	—	—
ID3	0%	—	—	0%	—	—	—	—	—	—	—
IDH1	0%	—	—	0%	5%	—	—	—	—	—	—
KRAS	0%	—	—	4%	—	—	—	—	—	—	—
MAP3K7	0%	—	—	0%	—	—	—	—	—	—	—
RHOA	0%	—	—	0%	4%	—	—	—	—	—	—
STAT5B	0%	—	—	2%	—	—	—	—	—	—	—
TRAF3	0%	—	1%	0%	—	—	—	—	—	—	—

Note: * They were next generation sequencing studies. Cell line data were excluded, and only DLBCL samples were considered for mutation frequencies.

1. Morin RD, Mendez-Lago M, Mungall AJ, Goya R, Mungall KL, Corbett RD, et al. Frequent mutation of histone-modifying genes in non-Hodgkin lymphoma. Nature. 2011;476:298-303.

2. Pasqualucci L, Trifonov V, Fabbri G, Ma J, Rossi D, Chiarenza A, et al. Analysis of the coding genome of diffuse large B-cell lymphoma. Nat Genet. 2011;43:830-7.

3. Lohr JG, Stojanov P, Lawrence MS, Auclair D, Chapuy B, Sougnez C, et al. Discovery and prioritization of somatic mutations in diffuse large B-cell lymphoma (DLBCL) by whole-exome sequencing. Proc Natl Acad Sci U S A. 2012;109:3879-84.

4. Zhang J, Grubor V, Love CL, Banerjee A, Richards KL, Mieczkowski PA, et al. Genetic heterogeneity of diffuse large B-cell lymphoma. Proc Natl Acad Sci U S A. 2013;110:1398-403.

5. Mottok A, Renne C, Seifert M, Oppermann E, Bechstein W, Hansmann ML, et al. Inactivating SOCS1 mutations are caused by aberrant somatic hypermutation and restricted to a subset of B-cell lymphoma entities. Blood. 2009;114:4503-6.

6. Schif B, Lennerz JK, Kohler CW, Bentink S, Kreuz M, Melzner I, et al. SOCS1 mutation subtypes predict divergent outcomes in diffuse large B-Cell lymphoma (DLBCL) patients. Oncotarget. 2013;4:35-47.

7. Arcaini L, Rossi D, Lucioni M, Nicola M, Bruscaggin A, Fiaccadori V, et al. The NOTCH pathway is recurrently mutated in diffuse large B-cell lymphoma associated with hepatitis C virus infection. Haematologica. 2015;100:246-52.

8. Asmar F, Punj V, Christensen J, Pedersen MT, Pedersen A, Nielsen AB, et al. Genome-wide profiling identifies a DNA methylation signature that associates with TET2 mutations in diffuse large B-cell lymphoma. Haematologica. 2013;98:1912-20.

9. Quivoron C, Couronne L, Della VV, Lopez CK, Plo I, Wagner-Ballon O, et al. TET2 inactivation results in pleiotropic hematopoietic abnormalities in mouse and is a recurrent event during human lymphomagenesis. Cancer Cell. 2011;20:25-38.

10. Trinh DL, Scott DW, Morin RD, Mendez-Lago M, An J, Jones SJ, et al. Analysis of FOXO1 mutations in diffuse large B-cell lymphoma. Blood. 2013;121:3666-74.

Table S3 List of mutations identified in *DDX3X* by targeted sequencing

Sample	Gene	RefSeq	Chromosome	Position	Reference allele	Alteration allele	Mutation type	cDNA change	AA Change	Alteration frequency	Read-depth
10	*DDX3X*	NM_001356	chrX	41204450	A	C	Missense	c.A1043C	p.E348A	0.096926714	846
24	*DDX3X*	NM_001356	chrX	41204703	G	A	Missense	c.G1217A	p.G406E	0.215231788	906
26	*DDX3X*	NM_001356	chrX	41203332	T	C	Missense	c.T815C	p.L272S	0.812658228	395
84	*DDX3X*	NM_001356	chrX	41204476	T	G	Missense	c.T1069G	p.F357V	0.32032032	999
87	*DDX3X*	NM_001356	chrX	41205629	G	A	Missense	c.G1463A	p.R488H	0.859832636	478
63	*DDX3X*	NM_001356	chrX	41202606	T	C	Splice site	c.679+2T>C	NA	0.113367174	591

Abbreviations: AA, amino acid; NA, not available.

Table S4 Mutations identified in pathway genes by targeted sequencing

Sample	Gene	RefSeq	Chromosome	Position	Reference allele	Alteration allele	Mutation type	cDNA change	AA change	Alteration frequency	Read depth
1	*CD79A*	NM_001783	chr19	42384801	A	C	Missense	c.A563C	p.Y188S	0.61827957	186
27	*CD79A*	NM_001783	chr19	42384800	T	G	Missense	c.T562G	p.Y188D	0.22429907	214
1	*CD79B*	NM_001039933	chr17	62006606	C	G	Missense	c.G673C	p.E225Q	0.45898004	451
1	*CD79B*	NM_001039933	chr17	62006666	T	A	Missense	c.A613T	p.T205S	0.43841336	479
1	*CD79B*	NM_001039933	chr17	62006804	T	C	Missense	c.A584G	p.H195R	0.50455927	329
16	*CD79B*	NM_001039933	chr17	62007135	C	G	Missense	c.G547C	p.D183H	0.08402204	726
18	*CD79B*	NM_001039933	chr17	62006799	A	C	Missense	c.T589G	p.Y197D	0.37265918	534
23	*CD79B*	NM_001039933	chr17	62006796	CGTAG	C	Frameshift deletion	c.588_591del	p.T196fs	0.11578947	475
25	*CD79B*	NM_001039933	chr17	62006799	A	G	Missense	c.T589C	p.Y197H	0.40480591	541
33	*CD79B*	NM_001039933	chr17	62006799	A	T	Missense	c.T589A	p.Y197N	0.25128205	390
37	*CD79B*	NM_001039933	chr17	62006798	T	C	Missense	c.A590G	p.Y197C	0.48341232	211
37	*CD79B*	NM_001039933	chr17	62006807	T	C	Missense	c.A581G	p.D194G	0.47524752	202
39	*CD79B*	NM_001039933	chr17	62006836	C	T	Splice site	553-1G>A	NA	0.22682927	410
53	*CD79B*	NM_001039933	chr17	62006798	T	G	Missense	c.A590C	p.Y197S	0.16302187	503
53	*CD79B*	NM_001039933	chr17	62006817	T	C	Missense	c.A571G	p.M191V	0.15686275	459
54	*CD79B*	NM_001039933	chr17	62006799	A	G	Missense	c.T589C	p.Y197H	0.85530547	311
61	*CD79B*	NM_001039933	chr17	62006798	T	A	Missense	c.A590T	p.Y197F	0.44803695	433
63	*CD79B*	NM_001039933	chr17	62006616	C	G	Missense	c.G663C	p.W221C	0.19575856	613
74	*CD79B*	NM_001039933	chr17	62008737	C	G	Missense	c.G79C	p.V27L	0.34496124	774
88	*CD79B*	NM_001039933	chr17	62006836	C	A	Splice site	c.553-1G>T	NA	0.29787234	329
92	*CD79B*	NM_001039933	chr17	62006680	A	C	Missense	c.T599G	p.L200R	0.25086306	869
92	*CD79B*	NM_001039933	chr17	62008737	C	T	Missense	c.G79A	p.V27I	0.35180055	722
92	*CD79B*	NM_001039933	chr17	62008750	T	G	Splice site	c.68-2A>C	NA	0.34104938	648
93	*CD79B*	NM_001039933	chr17	62006680	A	C	Missense	c.T599G	p.L200R	0.48508946	503
96	*CD79B*	NM_001039933	chr17	62006798	T	C	Missense	c.A590G	p.Y197C	0.69432314	458
23	*BTK*	NM_001287344	chrX	100630250	C	T	Missense	c.G125A	p.S42N	0.24092409	303
30	*BTK*	NM_001287344	chrX	100613402	TGACGTATCACCCCTTGAGGGTCCCTGAAGAAGTGGATGCTTAGTCA	T	Frameshift deletion	c.1077_1099del	p.G359fs	0.14692982	456
83	*BTK*	NM_001287344	chrX	100630254	C	T	Missense	c.G121A	p.E41K	0.34414414	555
17	*CARD11*	NM_032415	chr7	2983886	T	G	Missense	c.A644C	p.K215T	0.55855856	999
20	*CARD11*	NM_032415	chr7	2977603	A	C	Missense	c.T1081G	p.Y361D	0.42988506	870
24	*CARD11*	NM_032415	chr7	2972204	G	C	Missense	c.C1535G	p.S512C	0.556	1000
60	*CARD11*	NM_032415	chr7	2983885	C	G	Missense	c.G645C	p.K215N	0.197	1000
65	*CARD11*	NM_032415	chr7	2977584	G	A	Missense	c.C1100T	p.T367M	0.38386648	719
77	*CARD11*	NM_032415	chr7	2962890	C	T	Missense	c.G2018A	p.G673D	0.40439276	774
79	*CARD11*	NM_032415	chr7	2979381	A	C	Splice site	c.864+2T>G	NA	0.39899833	599
82	*CARD11*	NM_032415	chr7	2962792	G	A	Missense	c.C2116T	p.L706F	0.48028674	558
83	*CARD11*	NM_032415	chr7	2979435	C	T	Missense	c.G812A	p.R271Q	0.23923924	999
83	*CARD11*	NM_032415	chr7	2979495	A	G	Missense	c.T752C	p.L251P	0.24224224	999
83	*CARD11*	NM_032415	chr7	2985468	A	T	Missense	c.T343A	p.F115I	0.24481328	964
94	*CARD11*	NM_032415	chr7	2968244	G	A	Missense	c.C1742T	p.A581V	0.48050314	795
63	*MALT1*	NM_006785	chr18	56409112	A	G	Missense	c.A1619G	p.H540R	0.08643815	671
82	*MALT1*	NM_006785	chr18	56338904	C	T	Missense	c.C29T	p.A10V	0.58974359	39
82	*MALT1*	NM_006785	chr18	56363613	T	C	Missense	c.T392C	p.V131A	0.17156863	204
82	*MALT1*	NM_006785	chr18	56414849	T	A	Nonsense	c.T2250A	p.Y750X	0.426	1000
82	*MALT1*	NM_006785	chr18	56363596	A	T	Splice site	c.377-2A>T	NA	0.1512605	119
24	*BCL10*	NM_003921	chr1	85733572	AG	A	Frameshift deletion	c.439delC	p.L147X	0.22808989	890
50	*BCL10*	NM_003921	chr1	85736463	T	C	Missense	c.A184G	p.R62G	0.27755511	998
52	*BCL10*	NM_003921	chr1	85733392	AG	A	Frameshift deletion	c.619delC	p.L207fs	0.27653061	980
55	*BCL10*	NM_003921	chr1	85733594	C	A	Nonsense	c.G418T	p.E140X	0.46740859	629
65	*BCL10*	NM_003921	chr1	85733329	C	T	Missense	c.G683A	p.R228H	0.38812785	876
8	*PRKCB*	NM_002738	chr16	24183630	G	A	Missense	c.G1279A	p.D427N	0.40574506	557
27	*PRKCB*	NM_002738	chr16	24135291	A	G	Missense	c.A1054G	p.S352G	0.204	1000
27	*PRKCB*	NM_002738	chr16	24196802	G	A	Missense	c.G1636A	p.E546K	0.16090584	839
60	*PRKCB*	NM_002738	chr16	24231406	C	T	Missense	c.C1988T	p.T663I	0.28248588	885
68	*PRKCB*	NM_002738	chr16	23847659	G	C	Missense	c.G163C	p.D55H	0.13043478	736
75	*PRKCB*	NM_002738	chr16	24226023	T	G	Missense	c.T1908G	p.H636Q	0.31530612	980
1	*TCF3*	NM_001136139	chr19	1646419	G	A	Missense	c.C80T	p.P27L	0.98538012	342
7	*TCF3*	NM_001136139	chr19	1622210	T	C	Missense	c.A665G	p.H222R	0.48691099	191

51	*TCF3*	NM_001136139	chr19	1612366	A	T	Missense	c.T1653A	p.N551K	0.20143027	839
74	*TCF3*	NM_003200	chr19	1615283	C	G	Splice site	c.1822+1G>C	NA	0.66580311	386
3	*MYD88*	NM_002468	chr3	38182641	T	C	Missense	c.794T>C	p.L265P	0.77741935	930
4	*MYD88*	NM_002468	chr3	38182292	G	A	Missense	c.G728A	p.S243N	0.1516035	686
18	*MYD88*	NM_002468	chr3	38182032	C	G	Missense	c.C656G	p.S219C	0.65265265	999
44	*MYD88*	NM_002468	chr3	38182641	T	C	Missense	c.794T>C	p.L265P	0.58758759	999
53	*MYD88*	NM_002468	chr3	38182641	T	C	Missense	c.794T>C	p.L265P	0.124	1000
54	*MYD88*	NM_002468	chr3	38182641	T	C	Missense	c.794T>C	p.L265P	0.4184953	638
58	*MYD88*	NM_002468	chr3	38182292	G	A	Missense	c.G728A	p.S243N	0.45422535	852
59	*MYD88*	NM_001172567	chr3	38182073	C	T	Missense	c.C697T	p.P233S	0.51439299	799
61	*MYD88*	NM_002468	chr3	38182641	T	C	Missense	c.794T>C	p.L265P	0.39033457	807
67	*MYD88*	NM_002468	chr3	38181430	A	G	Missense	c.A443G	p.D148G	0.24742268	679
71	*MYD88*	NM_002468	chr3	38182292	G	A	Missense	c.G728A	p.S243N	0.22238806	670
73	*MYD88*	NM_002468	chr3	38182641	T	C	Missense	c.794T>C	p.L265P	0.6242485	998
75	*MYD88*	NM_002468	chr3	38182259	T	C	Missense	c.T695C	p.M232T	0.828	1000
77	*MYD88*	NM_002468	chr3	38182641	T	C	Missense	c.794T>C	p.L265P	0.36499466	937
78	*MYD88*	NM_002468	chr3	38182641	T	C	Missense	c.794T>C	p.L265P	0.10079576	754
80	*MYD88*	NM_002468	chr3	38182641	T	C	Missense	c.794T>C	p.L265P	0.33333333	990
85	*MYD88*	NM_002468	chr3	38182292	G	A	Missense	c.G728A	p.S243N	0.382	1000
86	*MYD88*	NM_002468	chr3	38182641	T	C	Missense	c.794T>C	p.L265P	0.76203576	727
89	*MYD88*	NM_002468	chr3	38182641	T	C	Missense	c.794T>C	p.L265P	0.47484909	497
92	*MYD88*	NM_002468	chr3	38182641	T	C	Missense	c.794T>C	p.L265P	0.31034483	870
13	*TNFAIP3*	NM_006290	chr6	138201292	C	G	Missense	c.C1991G	p.T664S	0.51680672	952
18	*TNFAIP3*	NM_006290	chr6	138199898	G	T	Missense	c.G1316T	p.R439L	0.76744186	473
19	*TNFAIP3*	NM_006290	chr6	138192661	T	A	Splice site	c.295+2T>A	NA	0.32890365	903
42	*TNFAIP3*	NM_006290	chr6	138198327	T	G	Nonsense	c.T920G	p.L307X	0.1521511	953
50	*TNFAIP3*	NM_006290	chr6	138192500	TAC	T	Frameshift deletion	c.137_138del	p.Y46fs	0.34455959	772
63	*TNFAIP3*	NM_006290	chr6	138199944	TG	T	Frameshift deletion	c.1363delG	p.G455fs	0.46687211	649
72	*TNFAIP3*	NM_006290	chr6	138196937	G	A	Missense	c.G599A	p.C200Y	0.45682731	996
63	*TRAF2*	NM_021138	chr9	139794078	A	C	Missense	c.A221C	p.H74P	0.28211009	872
63	*TRAF2*	NM_021138	chr9	139794896	G	A	Missense	c.G290A	p.R97H	0.27731092	476
2	*TRAF5*	NM_004619	chr1	211527763	CAG	C	Frameshift deletion	c.231_232del	p.T77fs	0.45183888	571
15	*TRAF5*	NM_004619	chr1	211534057	A	G	Missense	c.A557G	p.N186S	0.572	1000
32	*IKBKB*	NM_001556	chr8	42179896	G	A	Missense	c.G1870A	p.E624K	0.26394558	735
27	*TNFRSF11A*	NM_003839	chr18	60021678	C	T	Missense	c.C338T	p.A113V	0.22285714	350
26	*BIRC3*	NM_001165	chr11	102207742	T	C	Missense	c.T1724C	p.L575P	0.35930736	924
55	*BIRC3*	NM_001165	chr11	102207510	T	A	Nonsense	c.T1599A	p.Y533X	0.28526316	950
60	*BIRC3*	NM_001165	chr11	102195756	ATTAC	A	Frameshift deletion	c.517_520del	p.L173fs	0.24855491	865
80	*BIRC3*	NM_001165	chr11	102206864	A	G	Missense	c.A1492G	p.T498A	0.527	1000
26	*MAP3K14*	NM_003954	chr17	43344938	G	A	Missense	c.C2161T	p.P721S	0.46892655	531
5	*NFKBIA*	NM_020529	chr14	35871687	T	TG	Frameshift insertion	c.818dupC	p.T273fs	0.34229576	967
5	*NFKBIA*	NM_020529	chr14	35873711	TTGACCATC	T	Frameshift deletion	c.132_139del	p.Q44fs	0.07588358	962
38	*NFKBIA*	NM_020529	chr14	35873643	G	C	Missense	c.C208G	p.L70V	0.37325905	359
57	*NFKBIA*	NM_020529	chr14	35873643	G	C	Missense	c.C208G	p.L70V	0.08351648	455
63	*NFKBIA*	NM_020529	chr14	35873801	CG	C	Frameshift deletion	c.49delC	p.R17fs	0.23762376	707
5	*NOTCH1*	NM_017617	chr9	139391169	GAGGGGGCCTGTGTGCTC	G	Frameshift deletion	c.7005_7021del	p.L2335fs	0.29573935	399
15	*NOTCH1*	NM_017617	chr9	139402576	C	T	Missense	c.G3341A	p.R1114H	0.54366812	458
21	*NOTCH1*	NM_017617	chr9	139402523	G	A	Missense	c.C3394T	p.R1132C	0.53564547	519
29	*NOTCH1*	NM_017617	chr9	139413048	C	T	Missense	c.G1094A	p.R365H	0.45707657	431
44	*NOTCH1*	NM_017617	chr9	139403458	C	T	Missense	c.G3035A	p.G1012D	0.48780488	246
63	*NOTCH1*	NM_017617	chr9	139390734	G	GA	Frameshift insertion	c.7456dupT	p.S2486fs	0.20465116	645
74	*NOTCH1*	NM_017617	chr9	139391593	C	T	Missense	c.G6598A	p.V2200M	0.51290685	891
79	*NOTCH1*	NM_017617	chr9	139400212	G	A	Missense	c.C4136T	p.T1379M	0.40174672	458
1	*NOTCH2*	NM_024408	chr1	120458147	G	A	Nonsense	c.C7198T	p.R2400X	0.4637931	580
9	*NOTCH2*	NM_024408	chr1	120458161	G	C	Nonsense	c.C7184G	p.S2395X	0.38345865	798
57	*NOTCH2*	NM_024408	chr1	120539664	C	G	Missense	c.G707C	p.C236S	0.27444052	849
62	*NOTCH2*	NM_024408	chr1	120548153	G	A	Missense	c.C214T	p.R72C	0.128	1000
71	*NOTCH2*	NM_024408	chr1	120458435	TG	T	Frameshift deletion	c.6909delC	p.P2303fs	0.14652015	546
83	*NOTCH2*	NM_024408	chr1	120612014	C	A	Missense	c.G7T	p.A3S	0.15212982	986
86	*NOTCH2*	NM_024408	chr1	120480033	T	A	Missense	c.A3394T	p.T1132S	0.36363636	495
92	*NOTCH2*	NM_024408	chr1	120612014	C	A	Missense	c.G7T	p.A3S	0.11875694	901

96	*NOTCH2*	NM_024408	chr1	120612014	C	A	Missense	c.G7T	p.A3S	0.20666667	750
1	*SPEN*	NM_015001	chr1	16255496	C	T	Nonsense	c.C2761T	p.Q921X	0.93665158	884
4	*SPEN*	NM_015001	chr1	16254644	C	T	Nonsense	c.C1909T	p.R637X	0.1601467	818
10	*SPEN*	NM_015001	chr1	16202717	G	A	Missense	c.G425A	p.R142H	0.08138101	811
12	*SPEN*	NM_015001	chr1	16174588	G	A	Nonsense	c.G26A	p.W9X	0.71590909	352
16	*SPEN*	NM_015001	chr1	16259744	C	T	Nonsense	c.C7009T	p.R2337X	0.07880133	901
23	*SPEN*	NM_015001	chr1	16199421	C	T	Missense	c.C194T	p.S65L	0.50578339	951
27	*SPEN*	NM_015001	chr1	16258427	C	T	Nonsense	c.C5692T	p.R1898X	0.197	1000
29	*SPEN*	NM_015001	chr1	16259097	C	T	Missense	c.C6362T	p.P2121L	0.4959893	748
32	*SPEN*	NM_015001	chr1	16256716	ACTAT	A	Frameshift deletion	c.3982_3985del	p.L1328fs	0.23360656	976
48	*SPEN*	NM_015001	chr1	16245483	TGCTA	T	Frameshift deletion	c.1459_1462del	p.A487fs	0.37737738	999
48	*SPEN*	NM_015001	chr1	16255877	G	T	Nonsense	c.G3142T	p.E1048X	0.40781563	998
71	*SPEN*	NM_015001	chr1	16254929	C	T	Nonsense	c.C2194T	p.R732X	0.17313746	953
75	*SPEN*	NM_015001	chr1	16174611	G	T	Missense	c.G49T	p.V17L	0.64	525
75	*SPEN*	NM_015001	chr1	16256456	C	T	Nonsense	c.C3721T	p.R1241X	0.586	1000
84	*SPEN*	NM_015001	chr1	16255449	A	T	Missense	c.A2714T	p.N905I	0.52252252	999
87	*SPEN*	NM_015001	chr1	16245427	G	T	Missense	c.G1402T	p.D468Y	0.07738095	840
91	*SPEN*	NM_015001	chr1	16259097	C	T	Missense	c.C6362T	p.P2121L	0.44839068	901
96	*SPEN*	NM_015001	chr1	16259097	C	T	Missense	c.C6362T	p.P2121L	0.60275862	725
20	*FBXW7*	NM_033632	chr4	153247367	G	A	Nonsense	c.C1435T	p.R479X	0.6856492	439
37	*FBXW7*	NM_033632	chr4	153251907	G	A	Nonsense	c.C1099T	p.R367X	0.14842301	539
40	*FBXW7*	NM_033632	chr4	153245410	A	G	Missense	c.T1781C	p.L594P	0.094	1000
40	*FBXW7*	NM_033632	chr4	153247366	C	T	Missense	c.G1436A	p.R479Q	0.09268795	971
93	*FBXW7*	NM_033632	chr4	153249384	C	T	Missense	c.G1394A	p.R465H	0.27027027	999
21	*PIK3CA*	NM_006218	chr3	178943785	C	T	Missense	c.C2452T	p.R818C	0.33120341	939
35	*PIK3CA*	NM_006218	chr3	178928079	G	A	Missense	c.G1357A	p.E453K	0.23933403	961
68	*PIK3CD*	NM_005026	chr1	9780849	A	C	Missense	c.A1571C	p.Y524S	0.13	500
34	*PIK3CG*	NM_002649	chr7	106508037	G	A	Missense	c.G31A	p.V11M	0.49044586	157
49	*PIK3CG*	NM_002649	chr7	106509408	C	A	Missense	c.C1402A	p.L468M	0.657082	939
63	*PIK3CG*	NM_002649	chr7	106515234	G	A	Missense	c.G2377A	p.A793T	0.09834254	905
96	*PIK3CG*	NM_002649	chr7	106523575	C	G	Missense	c.C2727G	p.H909Q	0.488	1000
63	*PIK3R1*	NM_181523	chr5	67593274	A	T	Nonsense	c.A2020T	p.K674X	0.23207776	823
83	*PIK3R1*	NM_181523	chr5	67591125	T	C	Missense	c.T1718C	p.L573P	0.23823824	999
63	*PIK3R2*	NM_005027	chr19	18278059	T	C	Missense	c.T1679C	p.M560T	0.26204819	664
77	*PIK3R2*	NM_005027	chr19	18273072	G	A	Missense	c.G962A	p.S321N	0.50337079	445
63	*AKT2*	NM_001626	chr19	40748466	G	A	Missense	c.C416T	p.A139V	0.26742301	617
36	*AKT3*	NM_005465	chr1	243736225	TA	T	Splice site	c.819+2T>-	NA	0.25	192
54	*AKT3*	NM_005465	chr1	243736225	TA	T	Splice site	c.819+2T>-	NA	0.27513228	378
77	*AKT3*	NM_005465	chr1	243800989	A	G	Missense	c.T485C	p.V162A	0.3618202	901
1	*PTEN*	NM_000314	chr10	89624240	T	C	Missense	c.T14C	p.I5T	0.41795231	713
26	*PTEN*	NM_000314	chr10	89624275	C	T	Nonsense	c.C49T	p.Q17X	0.40723982	884
26	*PTEN*	NM_000314	chr10	89624287	T	C	Missense	c.T61C	p.F21L	0.41230068	878
26	*PTEN*	NM_000314	chr10	89685283	A	T	Nonsense	c.A178T	p.K60X	0.357022	591
42	*PTEN*	NM_000314	chr10	89653867	G	A	Splice site	c.164+1G>A	NA	0.15899582	717
55	*PTEN*	NM_000314	chr10	89624306	G	A	Splice site	c.79+1G>A	NA	0.42416452	778
75	*PTEN*	NM_000314	chr10	89653861	AGTAAG	A	Frameshift deletion	c.160_164del	p.V54fs	0.33495935	615
84	*PTEN*	NM_000314	chr10	89692851	T	G	Missense	c.T335G	p.L112R	0.31162325	998
84	*PTEN*	NM_000314	chr10	89692910	G	A	Missense	c.G394A	p.G132S	0.32532533	999
95	*PTEN*	NM_000314	chr10	89624287	T	A	Missense	c.T61A	p.F21I	0.24102564	975
11	*MTOR*	NM_004958	chr1	11190767	C	T	Missense	c.G5432A	p.R1811H	0.5	562
58	*MTOR*	NM_004958	chr1	11181402	C	A	Missense	c.G6834T	p.L2278F	0.46998723	783
63	*MTOR*	NM_004958	chr1	11182038	G	A	Missense	c.C6808T	p.R2270W	0.34080717	446
78	*MTOR*	NM_004958	chr1	11298609	T	C	Missense	c.A1852G	p.I618V	0.48996656	598
4	*SGK1*	NM_001143676	chr6	134495706	C	G	Missense	c.G380C	p.R127T	0.1828125	640
4	*SGK1*	NM_001143676	chr6	134495648	C	T	Splice site	c.437+1G>A	NA	0.18738404	539
5	*SGK1*	NM_005627	chr6	134495942	T	G	Missense	c.A4C	p.T2P	0.30745342	966
7	*SGK1*	NM_001143676	chr6	134494207	G	C	Missense	c.C788G	p.T263S	0.07674944	886
14	*SGK1*	NM_001143676	chr6	134496787	G	A	Nonsense	c.C13T	p.Q5X	0.42207792	154
30	*SGK1*	NM_001143676	chr6	134493891	C	T	Missense	c.G856A	p.E286K	0.08757396	845
30	*SGK1*	NM_001143676	chr6	134494418	C	G	Missense	c.G696C	p.K232N	0.12363239	914
30	*SGK1*	NM_001143676	chr6	134495170	C	G	Missense	c.G486C	p.E162D	0.247851	698
30	*SGK1*	NM_001143676	chr6	134495650	G	A	Missense	c.C436T	p.H146Y	0.11972789	735
30	*SGK1*	NM_001143676	chr6	134495714	C	G	Missense	c.G372C	p.K124N	0.12903226	868
30	*SGK1*	NM_001143676	chr6	134495724	G	C	Missense	c.C362G	p.A121G	0.2627907	860
30	*SGK1*	NM_005627	chr6	134495884	A	T	Missense	c.T62A	p.V21E	0.15106117	801
30	*SGK1*	NM_001143676	chr6	134495648	C	G	Splice site	c.437+1G>C	NA	0.26849315	730
42	*SGK1*	NM_001143676	chr6	134495159	G	A	Missense	c.C497T	p.A166V	0.13195343	773
46	*SGK1*	NM_001143676	chr6	134493361	G	T	Missense	c.C1041A	p.S347R	0.40186916	642
46	*SGK1*	NM_001143676	chr6	134493449	A	T	Nonsense	c.T953A	p.L318X	0.30932203	472

46	*SGK1*	NM_001143676	chr6	134495147	G	A	Missense	c.C509T	p.P170L	0.36347197	553
46	*SGK1*	NM_001143676	chr6	134495211	C	T	Missense	c.G445A	p.V149I	0.39926063	541
46	*SGK1*	NM_001143676	chr6	134495650	G	A	Missense	c.C436T	p.H146Y	0.5631068	206
46	*SGK1*	NM_001143676	chr6	134495652	T	A	Missense	c.A434T	p.K145I	0.56038647	207
46	*SGK1*	NM_001143676	chr6	134495653	T	A	Nonsense	c.A433T	p.K145X	0.56038647	207
46	*SGK1*	NM_001143676	chr6	134495655	C	T	Missense	c.G431A	p.C144Y	0.56730769	208
46	*SGK1*	NM_001143676	chr6	134495659	C	T	Missense	c.G427A	p.A143T	0.57603687	217
46	*SGK1*	NM_001143676	chr6	134495706	C	G	Missense	c.G380C	p.R127T	0.4470339	472
46	*SGK1*	NM_001143678	chr6	134496758	C	G	Missense	c.G42C	p.E14D	0.38888889	126
46	*SGK1*	NM_001143676	chr6	134495648	C	G	Splice site	c.437+1G>C	NA	0.5631068	206
46	*SGK1*	NM_001143676	chr6	134495725	C	G	Splice site	c.362-1G>C	NA	0.42802303	521
48	*SGK1*	NM_001143676	chr6	134495183	G	A	Missense	c.C473T	p.P158L	0.4352518	834
59	*SGK1*	NM_001143676	chr6	134495150	G	C	Missense	c.C506G	p.S169C	0.51554828	611
59	*SGK1*	NM_001143676	chr6	134495664	G	C	Missense	c.C422G	p.S141C	0.16185897	624
67	*SGK1*	NM_001143676	chr6	134495650	G	A	Missense	c.C436T	p.H146Y	0.29137931	580
79	*SGK1*	NM_001143676	chr6	134495724	G	C	Missense	c.C362G	p.A121G	0.37003058	981
92	*SGK1*	NM_001143676	chr6	134492047	T	C	Missense	c.A1330G	p.I444V	0.70841889	487
63	*JAK1*	NM_002227	chr1	65323452	C	T	Missense	c.G1345A	p.A449T	0.25434116	979
40	*JAK2*	NM_004972	chr9	5078333	G	C	Missense	c.G2020C	p.V674L	0.563	1000
61	*JAK2*	NM_004972	chr9	5090802	G	A	Missense	c.G2950A	p.V984M	0.45730994	855
65	*JAK2*	NM_004972	chr9	5064964	C	G	Missense	c.C1138G	p.H380D	0.452	1000
78	*JAK2*	NM_004972	chr9	5126714	A	G	Missense	c.A3322G	p.N1108D	0.47731092	595
1	*JAK3*	NM_000215	chr19	17954665	C	A	Missense	c.G229T	p.D77Y	0.68205805	758
17	*JAK3*	NM_000215	chr19	17953136	C	T	Missense	c.G850A	p.G284R	0.46634615	208
4	*STAT3*	NM_139276	chr17	40475060	CCTT	C	Nonframeshift deletion	c.1847_1849del	p.616_617c	0.14666667	600
12	*STAT3*	NM_139276	chr17	40475070	T	G	Missense	c.A1840C	p.S614R	0.11818182	660
30	*STAT3*	NM_139276	chr17	40475060	CCTT	C	Nonframeshift deletion	c.1847_1849del	p.616_617c	0.20887246	541
55	*STAT3*	NM_139276	chr17	40485708	C	A	Missense	c.G1032T	p.Q344H	0.33183857	669
55	*STAT3*	NM_139276	chr17	40486035	G	T	Missense	c.C830A	p.T277N	0.32832833	999
63	*STAT3*	NM_139276	chr17	40468878	C	CG	Frameshift insertion	c.2185dupC	p.R729fs	0.20754717	848
63	*STAT3*	NM_139276	chr17	40481583	A	T	Missense	c.T1222A	p.F408I	0.16985951	783
68	*STAT3*	NM_139276	chr17	40474420	C	A	Missense	c.G1981T	p.D661Y	0.115	1000
83	*STAT3*	NM_139276	chr17	40481465	T	A	Missense	c.A1244T	p.E415V	0.29445074	883
83	*STAT3*	NM_139276	chr17	40481466	C	A	Nonsense	c.G1243T	p.E415X	0.29218573	883
63	*STAT5A*	NM_003152	chr17	40461415	A	G	Missense	c.A2135G	p.D712G	0.23324397	746
63	*STAT5A*	NM_003152	chr17	40461476	G	T	Missense	c.G2196T	p.Q732H	0.07651007	745
12	*STAT6*	NM_003153	chr12	57490463	C	A	Missense	c.G2436T	p.E812D	0.49746193	591
24	*STAT6*	NM_003153	chr12	57496661	T	C	Missense	c.A1256G	p.D419G	0.22945205	876
49	*STAT6*	NM_003153	chr12	57498330	C	G	Missense	c.G1129C	p.E377Q	0.42914172	501
50	*STAT6*	NM_003153	chr12	57496661	T	G	Missense	c.A1256C	p.D419A	0.45205479	803
50	*STAT6*	NM_003153	chr12	57496668	T	A	Missense	c.A1249T	p.N417Y	0.45646438	758
60	*STAT6*	NM_003153	chr12	57496661	T	C	Missense	c.A1256G	p.D419G	0.41255144	972
79	*STAT6*	NM_003153	chr12	57496661	T	G	Missense	c.A1256C	p.D419A	0.35907723	997
95	*STAT6*	NM_003153	chr12	57496661	T	G	Missense	c.A1256C	p.D419A	0.63681592	804
4	*SOCS1*	NM_003745	chr16	11349154	T	C	Missense	c.A182G	p.H61R	0.18819188	271
4	*SOCS1*	NM_003745	chr16	11349329	C	T	Missense	c.G7A	p.A3T	0.1732852	277
5	*SOCS1*	NM_003745	chr16	11348988	G	C	Missense	c.C348G	p.S116R	0.314367	703
8	*SOCS1*	NM_003745	chr16	11348988	G	C	Missense	c.C348G	p.S116R	0.48717949	429
15	*SOCS1*	NM_003745	chr16	11349184	CCGGGGGCCGGGGCCGGGACCGCGGGGCACGGCCGCGGGCGCG	C	Nonframeshift deletion	c.110_151del	p.37_51del	0.2027972	286
21	*SOCS1*	NM_003745	chr16	11348807	G	C	Missense	c.C529G	p.L177V	0.40206186	582
21	*SOCS1*	NM_003745	chr16	11349146	A	C	Missense	c.T190G	p.Y64D	0.375	344
24	*SOCS1*	NM_003745	chr16	11349287	C	T	Missense	c.G49A	p.A17T	0.26255708	438
30	*SOCS1*	NM_003745	chr16	11348762	C	T	Missense	c.G574A	p.A192T	0.12837838	444
30	*SOCS1*	NM_003745	chr16	11348807	G	C	Missense	c.C529G	p.L177V	0.47165533	441
30	*SOCS1*	NM_003745	chr16	11348853	C	A	Missense	c.G483T	p.M161I	0.12258065	465
30	*SOCS1*	NM_003745	chr16	11348866	G	C	Missense	c.C470G	p.A157G	0.15065502	458
30	*SOCS1*	NM_003745	chr16	11348880	C	G	Missense	c.G456C	p.E152D	0.13943355	459
30	*SOCS1*	NM_003745	chr16	11348888	G	C	Missense	c.C448G	p.L150V	0.49689441	483
30	*SOCS1*	NM_003745	chr16	11348908	C	G	Missense	c.G428C	p.S143T	0.09381238	501
30	*SOCS1*	NM_003745	chr16	11348988	G	C	Missense	c.C348G	p.S116R	0.53320683	527
30	*SOCS1*	NM_003745	chr16	11349199	G	A	Missense	c.C137T	p.P46L	0.36075949	158
30	*SOCS1*	NM_003745	chr16	11349287	C	G	Missense	c.G49C	p.A17P	0.43283582	201
30	*SOCS1*	NM_003745	chr16	11349301	G	A	Missense	c.C35T	p.A12V	0.43349754	203

37	*SOCS1*	NM_003745	chr16	11349077	G	A	Missense	c.C259T	p.H87Y	0.18560606	264
38	*SOCS1*	NM_003745	chr16	11348962	C	G	Missense	c.G374C	p.S125T	0.24683544	474
38	*SOCS1*	NM_003745	chr16	11349047	G	C	Missense	c.C289G	p.P97A	0.21967213	305
38	*SOCS1*	NM_003745	chr16	11349103	C	T	Missense	c.G233A	p.G78E	0.16666667	180
38	*SOCS1*	NM_003745	chr16	11349112	T	C	Missense	c.A224G	p.D75G	0.16363636	165
38	*SOCS1*	NM_003745	chr16	11349139	C	G	Missense	c.G197C	p.R66P	0.23333333	150
38	*SOCS1*	NM_003745	chr16	11349199	G	A	Missense	c.C137T	p.P46L	0.17021277	141
38	*SOCS1*	NM_003745	chr16	11349332	C	G	Missense	c.G4C	p.V2L	0.20467836	171
39	*SOCS1*	NM_003745	chr16	11348807	G	C	Missense	c.C529G	p.L177V	0.237691	589
39	*SOCS1*	NM_003745	chr16	11349093	C	T	Nonsense	c.G243A	p.W81X	0.23737374	396
39	*SOCS1*	NM_003745	chr16	11349329	C	T	Missense	c.G7A	p.A3T	0.20168067	357
42	*SOCS1*	NM_003745	chr16	11348725	A	G	Missense	c.T611C	p.L204P	0.1023766	547
42	*SOCS1*	NM_003745	chr16	11348749	A	G	Missense	c.T587C	p.L196P	0.1130742	566
42	*SOCS1*	NM_003745	chr16	11348988	G	C	Missense	c.C348G	p.S116R	0.11741425	758
42	*SOCS1*	NM_003745	chr16	11349018	G	C	Missense	c.C318G	p.S106R	0.15542522	682
42	*SOCS1*	NM_003745	chr16	11349077	G	A	Missense	c.C259T	p.H87Y	0.08552632	456
42	*SOCS1*	NM_003745	chr16	11349265	T	G	Missense	c.A71C	p.E24A	0.078125	320
48	*SOCS1*	NM_003745	chr16	11348706	C	A	Missense	c.G630T	p.Q210H	0.39095315	619
48	*SOCS1*	NM_003745	chr16	11348831	G	A	Missense	c.C505T	p.R169C	0.3803056	589
48	*SOCS1*	NM_003745	chr16	11348971	C	T	Missense	c.G365A	p.G122E	0.34085779	886
48	*SOCS1*	NM_003745	chr16	11348972	C	A	Nonsense	c.G364T	p.G122X	0.34308211	889
48	*SOCS1*	NM_003745	chr16	11349093	C	G	Missense	c.G243C	p.W81C	0.37772926	458
48	*SOCS1*	NM_003745	chr16	11349206	C	T	Missense	c.G130A	p.A44T	0.43333333	330
48	*SOCS1*	NM_003745	chr16	11349263	G	C	Missense	c.C73G	p.P25A	0.31042654	422
50	*SOCS1*	NM_003745	chr16	11348836	CGCAGCG GG	C	Frameshift deletion	c.492_499del	p.A164fs	0.24582339	419
50	*SOCS1*	NM_003745	chr16	11348845	G	A	Missense	c.C491T	p.A164V	0.24475524	429
51	*SOCS1*	NM_003745	chr16	11349214	G	A	Missense	c.C122T	p.P41L	0.22352941	340
57	*SOCS1*	NM_003745	chr16	11349142	CG	C	Frameshift deletion	c.193delC	p.R65fs	0.09950249	201
58	*SOCS1*	NM_003745	chr16	11349082	C	T	Missense	c.G254A	p.S85N	0.43316832	404
58	*SOCS1*	NM_003745	chr16	11349334	A	C	Missense	c.T2G	p.M1R	0.46153846	286
58	*SOCS1*	NM_003745	chr16	11349335	T	C	Missense	c.A1G	p.M1V	0.44210526	285
60	*SOCS1*	NM_003745	chr16	11349163	A	G	Missense	c.T173C	p.F58S	0.24199288	281
60	*SOCS1*	NM_003745	chr16	11349329	C	T	Missense	c.G7A	p.A3T	0.23076923	260
63	*SOCS1*	NM_003745	chr16	11348813	G	A	Nonsense	c.C523T	p.Q175X	0.26666667	510
63	*SOCS1*	NM_003745	chr16	11348908	C	G	Missense	c.G428C	p.S143T	0.08049536	646
63	*SOCS1*	NM_003745	chr16	11348933	A	C	Missense	c.T403G	p.F135V	0.21604938	648
63	*SOCS1*	NM_003745	chr16	11348963	T	G	Missense	c.A373C	p.S125R	0.20858896	652
63	*SOCS1*	NM_003745	chr16	11348971	C	G	Missense	c.G365C	p.G122A	0.20094192	637
63	*SOCS1*	NM_003745	chr16	11348972	C	T	Missense	c.G364A	p.G122R	0.48657188	633
63	*SOCS1*	NM_003745	chr16	11348984	T	G	Missense	c.A352C	p.K118Q	0.22291022	646
63	*SOCS1*	NM_003745	chr16	11348989	C	T	Missense	c.G347A	p.S116N	0.27848101	632
63	*SOCS1*	NM_003745	chr16	11349241	G	A	Missense	c.C95T	p.S32L	0.22256098	328
63	*SOCS1*	NM_003745	chr16	11349301	G	C	Missense	c.C35G	p.A12G	0.22772277	303
63	*SOCS1*	NM_003745	chr16	11349324	G	T	Missense	c.C12A	p.H4Q	0.20357143	280
63	*SOCS1*	NM_003745	chr16	11349386	C	G	Splice site	NA	NA	0.23312883	163
67	*SOCS1*	NM_003745	chr16	11348889	C	G	Missense	c.G447C	p.E149D	0.23802164	647
70	*SOCS1*	NM_003745	chr16	11349033	G	C	Missense	c.C303G	p.F101L	0.27056277	462
70	*SOCS1*	NM_003745	chr16	11349146	A	T	Missense	c.T190A	p.Y64N	0.26587302	252
70	*SOCS1*	NM_003745	chr16	11349236	C	A	Missense	c.G100T	p.A34S	0.27272727	297
70	*SOCS1*	NM_003745	chr16	11349290	C	T	Missense	c.G46A	p.A16T	0.30111524	269
70	*SOCS1*	NM_003745	chr16	11349320	G	A	Nonsense	c.C16T	p.Q6X	0.30241935	248
81	*SOCS1*	NM_003745	chr16	11348977	G	A	Missense	c.C359T	p.A120V	0.46757679	586
85	*SOCS1*	NM_003745	chr16	11349327	TGC	T	Frameshift deletion	c.7_8del	p.A3fs	0.28928571	280
95	*SOCS1*	NM_003745	chr16	11349010	CGCTGGC G	C	Frameshift deletion	c.319_325del	p.R107fs	0.37683284	682
95	*SOCS1*	NM_003745	chr16	11348713	G	A	Missense	c.C623T	p.P208L	0.32409012	577
95	*SOCS1*	NM_003745	chr16	11348791	A	T	Missense	c.T545A	p.I182N	0.3037037	540
95	*SOCS1*	NM_003745	chr16	11348808	C	G	Missense	c.G528C	p.E176D	0.52427184	515
95	*SOCS1*	NM_003745	chr16	11348900	A	T	Missense	c.T436A	p.C146S	0.33135215	673
95	*SOCS1*	NM_003745	chr16	11348911	T	C	Missense	c.A425G	p.E142G	0.15285714	700
95	*SOCS1*	NM_003745	chr16	11348927	G	C	Missense	c.C409G	p.L137V	0.19066148	771
95	*SOCS1*	NM_003745	chr16	11348962	C	G	Missense	c.G374C	p.S125T	0.18397997	799
95	*SOCS1*	NM_003745	chr16	11349093	C	A	Missense	c.G243T	p.W81C	0.53741497	294
95	*SOCS1*	NM_003745	chr16	11349097	T	A	Missense	c.A239T	p.Y80F	0.30388693	283
95	*SOCS1*	NM_003745	chr16	11349099	G	C	Missense	c.C237G	p.F79L	0.31521739	276
95	*SOCS1*	NM_003745	chr16	11349328	G	A	Missense	c.C8T	p.A3V	0.18421053	228
95	*SOCS1*	NM_003745	chr16	11349332	C	T	Missense	c.G4A	p.V2I	0.45045045	222
95	*SOCS1*	NM_003745	chr16	11349333	C	T	Missense	c.G3A	p.M1I	0.4529148	223
30	*PTPN1*	NM_002827	chr20	49177964	G	A	Missense	c.G128A	p.R43Q	0.2978236	873

84	*MPL*	NM_005373	chr1	43812466	G	A	Missense	c.G1169A	p.R390H	0.32252836	617
81	*NRAS*	NM_002524	chr1	115256424	T	A	Missense	c.A287T	p.Y96F	0.49928469	699
42	*BRAF*	NM_004333	chr7	140434420	A	T	Missense	c.T2278A	p.Y760N	0.11447811	891
57	*BRAF*	NM_004333	chr7	140494242	G	A	Missense	c.C1006T	p.P336S	0.0907781	694
63	*BRAF*	NM_004333	chr7	140624476	C	A	Missense	c.G28T	p.G10C	0.12389381	113
70	*BRAF*	NM_004333	chr7	140550014	T	TA	Splice site	c.139-2->T	NA	0.22030238	463
1	*GNA13*	NM_006572	chr17	63052556	C	CAGGAT	Frameshift insertion	c.155_156ins ATCCT	p.L52fs	0.45733788	586
5	*GNA13*	NM_006572	chr17	63052631	C	G	Missense	c.G81C	p.Q27H	0.3693495	907
42	*GNA13*	NM_006572	chr17	63010508	CGTTT	C	Frameshift deletion	c.997_1000de l	p.K333fs	0.115	1000
63	*GNA13*	NM_006572	chr17	63010815	GT	G	Frameshift deletion	c.693delA	p.K231fs	0.276	1000
63	*GNA13*	NM_006572	chr17	63049800	C	T	Nonsense	c.G330A	p.W110X	0.24640884	905
68	*GNA13*	NM_006572	chr17	63052533	C	A	Missense	c.G179T	p.G60V	0.13846154	715
70	*GNA13*	NM_006572	chr17	63010926	CTTG	C	Nonframeshift deletion	c.580_582del	p.194_194c	0.26846847	555
84	*GNA13*	NM_006572	chr17	63052601	G	GCATTTG	Frameshift insertion	c.110_111ins AGATCGAC AAATG	p.C37_L38	0.20864865	925
84	*GNA13*	NM_006572	chr17	63049718	C	A	Nonsense	c.G412T	p.E138X	0.28986961	997
27	*S1PR2*	NM_004230	chr19	10334663	T	A	Missense	c.A919T	p.R307W	0.48711944	427
63	*S1PR2*	NM_004230	chr19	10334663	T	A	Missense	c.A919T	p.R307W	0.22580645	341
68	*S1PR2*	NM_004230	chr19	10334697	C	CA	Frameshift insertion	c.884dupT	p.L295fs	0.12860892	381
90	*S1PR2*	NM_004230	chr19	10334663	T	A	Missense	c.A919T	p.R307W	0.47683924	367
7	*ARID1A*	NM_006015	chr1	27087377	A	G	Missense	c.A1951G	p.M651V	0.44647887	710
14	*ARID1A*	NM_006015	chr1	27101442	C	A	Missense	c.C4724A	p.P1575Q	0.21791768	413
27	*ARID1A*	NM_006015	chr1	27059218	A	C	Missense	c.A1855C	p.T619P	0.4923913	920
35	*ARID1A*	NM_006015	chr1	27057892	T	TA	Frameshift insertion	c.1601dupA	p.Y534_P5	0.32522124	452
48	*ARID1A*	NM_006015	chr1	27023819	C	T	Nonsense	c.C925T	p.Q309X	0.35769231	260
68	*ARID1A*	NM_006015	chr1	27106237	C	G	Missense	c.C5848G	p.R1950G	0.517	1000
82	*ARID1A*	NM_006015	chr1	27056209	C	T	Missense	c.C1205T	p.S402L	0.45088161	794
84	*ARID1A*	NM_006015	chr1	27023476	CT	C	Frameshift deletion	c.583delT	p.Y195fs	0.32357247	683
84	*ARID1A*	NM_006015	chr1	27023540	T	C	Missense	c.T646C	p.Y216H	0.28688525	732
86	*ARID1A*	NM_006015	chr1	27023567	C	T	Missense	c.C673T	p.P225S	0.17391304	368
92	*ARID1A*	NM_006015	chr1	27023404	CCAA	C	Nonframeshift deletion	c.511_513del	p.171_171c	0.10970464	474
3	*CREBBP*	NM_004380	chr16	3830771	AT	A	Frameshift deletion	c.1784delA	p.H595fs	0.3408393	977
4	*CREBBP*	NM_004380	chr16	3843538	CTG	C	Frameshift deletion	c.1063_1064d el	p.Q355fs	0.15615616	666
4	*CREBBP*	NM_004380	chr16	3832701	C	G	Missense	c.G1557C	p.R519S	0.14823009	452
7	*CREBBP*	NM_004380	chr16	3788617	C	T	Missense	c.G4337A	p.R1446H	0.12723449	951
19	*CREBBP*	NM_004380	chr16	3788646	A	T	Missense	c.T4308A	p.S1436R	0.69169169	999
32	*CREBBP*	NM_004380	chr16	3789636	C	T	Missense	c.G4223A	p.C1408Y	0.37672584	507
42	*CREBBP*	NM_004380	chr16	3832706	T	C	Missense	c.A1552G	p.M518V	0.11755486	638
52	*CREBBP*	NM_004380	chr16	3788657	A	T	Missense	c.T4297A	p.Y1433N	0.37981859	882
56	*CREBBP*	NM_004380	chr16	3823913	G	A	Nonsense	c.C2302T	p.R768X	0.3875969	645
63	*CREBBP*	NM_004380	chr16	3900710	GA	G	Frameshift deletion	c.385delT	p.S129fs	0.25	576
72	*CREBBP*	NM_004380	chr16	3819324	T	C	Missense	c.A2911G	p.R971G	0.44615385	845
83	*CREBBP*	NM_004380	chr16	3808015	A	G	Missense	c.T3404C	p.L1135P	0.32817869	582
93	*CREBBP*	NM_004380	chr16	3786805	CCT	C	Frameshift deletion	c.4404_4405d el	p.T1468fs	0.28318584	791
8	*EP300*	NM_001429	chr22	41569735	G	T	Missense	c.G4726T	p.V1576L	0.50642202	545
18	*EP300*	NM_001429	chr22	41566522	T	G	Missense	c.T4399G	p.Y1467D	0.351	1000
50	*EP300*	NM_001429	chr22	41489052	G	GGCCT	Frameshift insertion	c.44_45insGC CT	p.R15fs	0.24946237	465
63	*EP300*	NM_001429	chr22	41566524	CA	C	Frameshift deletion	c.4402delA	p.K1468fs	0.23734533	889
63	*EP300*	NM_001429	chr22	41513611	G	A	Missense	c.G515A	p.G172D	0.267	1000
78	*EP300*	NM_001429	chr22	41565529	G	A	Missense	c.G4195A	p.D1399N	0.08652482	705
31	*DNMT3A*	NM_175629	chr2	25463588	C	T	Nonsense	c.G2094A	p.W698X	0.36096718	579
57	*DNMT3A*	NM_175629	chr2	25468133	G	A	Nonsense	c.C1543T	p.Q515X	0.47520661	484
7	*EZH2*	NM_004456	chr7	148508728	A	T	Missense	c.T1936A	p.Y646N	0.09882353	850
19	*EZH2*	NM_004456	chr7	148508728	A	T	Missense	c.T1936A	p.Y646N	0.33229167	960
24	*EZH2*	NM_004456	chr7	148508728	A	T	Missense	c.T1936A	p.Y646N	0.20168067	952
24	*EZH2*	NM_004456	chr7	148508799	G	C	Missense	c.C1865G	p.A622G	0.21372328	889
87	*EZH2*	NM_004456	chr7	148508727	T	C	Missense	c.A1937G	p.Y646C	0.44618834	892

64	*HDAC1*	NM_004964	chr1	32797709	G	A	Missense	c.G1238A	p.R413Q	0.46816479	267
68	*HDAC1*	NM_004964	chr1	32790085	G	T	Missense	c.G286T	p.V96F	0.155	1000
6	*HDAC4*	NM_006037	chr2	240036859	C	T	Missense	c.G1666A	p.V556M	0.6975	400
49	*HDAC4*	NM_006037	chr2	240061448	T	C	Missense	c.A910G	p.N304D	0.26666667	645
49	*HDAC4*	NM_006037	chr2	240111758	G	A	Missense	c.C110T	p.A37V	0.09259259	162
75	*HDAC4*	NM_006037	chr2	240098241	C	T	Missense	c.G358A	p.A120T	0.64011799	339
21	*HDAC7*	NM_015401	chr12	48187256	G	A	Missense	c.C1574T	p.S525F	0.30952381	168
21	*HDAC7*	NM_015401	chr12	48190923	A	C	Missense	c.T580G	p.S194A	0.38422392	786
53	*HDAC7*	NM_015401	chr12	48189035	C	T	Missense	c.G1216A	p.A406T	0.43793103	290
80	*HDAC7*	NM_015401	chr12	48187256	G	A	Missense	c.C1574T	p.S525F	0.16571429	175
80	*HDAC7*	NM_015401	chr12	48190798	A	G	Splice site	c.703+2T>C	NA	0.15467075	653
1	*HIST1H1C*	NM_005319	chr6	26056380	G	C	Missense	c.C277G	p.L93V	0.48376623	616
19	*HIST1H1C*	NM_005319	chr6	26056356	C	T	Missense	c.G301A	p.A101T	0.71881838	914
30	*HIST1H1C*	NM_005319	chr6	26056498	C	G	Missense	c.G159C	p.E53D	0.14824798	742
38	*HIST1H1C*	NM_005319	chr6	26056289	G	T	Missense	c.C368A	p.A123E	0.09323583	547
67	*HIST1H1C*	NM_005319	chr6	26056475	G	A	Missense	c.C182T	p.A61V	0.20653319	949
85	*HIST1H1C*	NM_005319	chr6	26056530	G	C	Missense	c.C127G	p.L43V	0.3488665	794
86	*HIST1H1C*	NM_005319	chr6	26056205	G	A	Missense	c.C452T	p.A151V	0.17410229	919
86	*HIST1H1C*	NM_005319	chr6	26056266	G	A	Missense	c.C391T	p.P131S	0.17978848	851
86	*HIST1H1C*	NM_005319	chr6	26056305	G	A	Missense	c.C352T	p.P118S	0.17059484	891
86	*HIST1H1C*	NM_005319	chr6	26056464	C	G	Missense	c.G193C	p.A65P	0.132	1000
92	*HIST1H1C*	NM_005319	chr6	26056419	T	G	Missense	c.A238C	p.I80L	0.52071669	893
93	*HIST1H1C*	NM_005319	chr6	26056323	C	T	Missense	c.G334A	p.A112T	0.11440108	743
1	*HIST1H1E*	NM_005321	chr6	26156850	A	T	Missense	c.A232T	p.S78C	0.39230769	390
1	*HIST1H1E*	NM_005321	chr6	26156937	C	T	Missense	c.C319T	p.L107F	0.36200717	279
6	*HIST1H1E*	NM_005321	chr6	26157105	G	A	Missense	c.G487A	p.A163T	0.29526462	359
7	*HIST1H1E*	NM_005321	chr6	26156929	C	T	Missense	c.C311T	p.S104F	0.09176471	425
12	*HIST1H1E*	NM_005321	chr6	26156811	G	A	Missense	c.G193A	p.A65T	0.378125	640
30	*HIST1H1E*	NM_005321	chr6	26156766	G	T	Missense	c.G148T	p.A50S	0.22363636	550
30	*HIST1H1E*	NM_005321	chr6	26156925	G	C	Missense	c.G307C	p.G103R	0.30635838	346
30	*HIST1H1E*	NM_005321	chr6	26156937	C	G	Missense	c.C319G	p.L107V	0.3088685	327
30	*HIST1H1E*	NM_005321	chr6	26157109	C	G	Missense	c.C491G	p.A164G	0.40069686	287
30	*HIST1H1E*	NM_005321	chr6	26157163	A	G	Missense	c.A545G	p.K182R	0.32236842	304
38	*HIST1H1E*	NM_005321	chr6	26156937	C	T	Missense	c.C319T	p.L107F	0.13173653	334
38	*HIST1H1E*	NM_005321	chr6	26157090	G	T	Missense	c.G472T	p.A158S	0.18589744	312
38	*HIST1H1E*	NM_005321	chr6	26157117	G	C	Missense	c.G499C	p.A167P	0.18575851	323
39	*HIST1H1E*	NM_005321	chr6	26156876	C	A	Missense	c.C258A	p.S86R	0.19601838	653
44	*HIST1H1E*	NM_005321	chr6	26157184	C	T	Missense	c.C566T	p.A189V	0.37888199	483
62	*HIST1H1E*	NM_005321	chr6	26156985	G	C	Missense	c.G367C	p.A123P	0.55907781	347
68	*HIST1H1E*	NM_005321	chr6	26156874	A	C	Missense	c.A256C	p.S86R	0.08846761	633
68	*HIST1H1E*	NM_005321	chr6	26156985	G	T	Missense	c.G367T	p.A123S	0.10684932	365
70	*HIST1H1E*	NM_005321	chr6	26156971	C	T	Missense	c.C353T	p.P118L	0.5046729	321
72	*HIST1H1E*	NM_005321	chr6	26157252	A	G	Missense	c.A634G	p.K212E	0.37516513	757
80	*HIST1H1E*	NM_005321	chr6	26156745	C	T	Missense	c.C127T	p.L43F	0.088	1000
85	*HIST1H1E*	NM_005321	chr6	26156935	A	G	Missense	c.A317G	p.K106R	0.45146727	443
86	*HIST1H1E*	NM_005321	chr6	26156926	G	T	Missense	c.G308T	p.G103V	0.17434211	608
86	*HIST1H1E*	NM_005321	chr6	26157012	G	C	Missense	c.G394C	p.A132P	0.13849765	426
86	*HIST1H1E*	NM_005321	chr6	26157099	C	T	Missense	c.C481T	p.P161S	0.18623482	494
91	*HIST1H1E*	NM_005321	chr6	26157187	A	G	Missense	c.A569G	p.K190R	0.13305613	481
92	*HIST1H1E*	NM_005321	chr6	26156985	G	C	Missense	c.G367C	p.A123P	0.17717718	333
76	*IDH2*	NM_002168	chr15	90633719	C	T	Missense	c.G365A	p.R122H	0.52089136	359
3	*KDM2B*	NM_032590	chr12	121877834	G	A	Missense	c.C3655T	p.R1219C	0.24383562	365
3	*KDM2B*	NM_032590	chr12	121880596	T	C	Missense	c.A2648G	p.N883S	0.37837838	148
5	*KDM2B*	NM_032590	chr12	121878978	G	A	Missense	c.C3343T	p.P1115S	0.50888325	788
23	*KDM2B*	NM_032590	chr12	121891060	G	A	Missense	c.C1822T	p.R608W	0.11904762	672
46	*KDM2B*	NM_032590	chr12	121880183	G	A	Missense	c.C3061T	p.R1021C	0.45989305	187
73	*KDM2B*	NM_032590	chr12	121880545	G	A	Missense	c.C2699T	p.A900V	0.55018587	269
90	*KDM2B*	NM_032590	chr12	122013710	C	T	Missense	c.G326A	p.R109Q	0.44062947	699
2	*KMT2C*	NM_170606	chr7	151970840	C	T	Missense	c.G962A	p.S321N	0.08608609	999
3	*KMT2C*	NM_170606	chr7	151859297	GAGA	G	Nonframeshift deletion	c.11362_11364del	p.3788_378	0.28059072	948
5	*KMT2C*	NM_170606	chr7	151860520	G	A	Missense	c.C10142T	p.P3381L	0.34	1000
5	*KMT2C*	NM_170606	chr7	151864263	G	C	Missense	c.C9718G	p.Q3240E	0.387	1000
16	*KMT2C*	NM_170606	chr7	151851136	G	A	Missense	c.C12235T	p.L4079F	0.47547548	999
25	*KMT2C*	NM_170606	chr7	151970840	C	T	Missense	c.G962A	p.S321N	0.083	1000
31	*KMT2C*	NM_170606	chr7	151875096	C	CT	Splice site	c.7443-1->A	NA	0.29787234	94
37	*KMT2C*	NM_170606	chr7	151962290	C	G	Missense	c.G1017C	p.K339N	0.1280683	937
42	*KMT2C*	NM_170606	chr7	152012286	T	C	Missense	c.A527G	p.N176S	0.53853854	999
48	*KMT2C*	NM_170606	chr7	151927050	AG	A	Frameshift deletion	c.2933delC	p.S978fs	0.13069909	987
53	*KMT2C*	NM_170606	chr7	151945124	T	C	Missense	c.A2395G	p.N799D	0.08708709	999
58	*KMT2C*	NM_170606	chr7	151874971	G	A	Missense	c.C7567T	p.P2523S	0.49190939	618

63	*KMT2C*	NM_170606	chr7	151845357	C	A	Missense	c.G13655T	p.S4552I	0.1718107	972
78	*KMT2C*	NM_170606	chr7	152132717	C	T	Missense	c.G155A	p.R52K	0.66666667	393
90	*KMT2C*	NM_170606	chr7	151935841	A	G	Missense	c.T2603C	p.I868T	0.401	1000
91	*KMT2C*	NM_170606	chr7	152132801	G	A	Missense	c.C71T	p.A24V	0.47658402	363
1	*KMT2D*	NM_003482	chr12	49418461	AAT	A	Frameshift deletion	c.15950_15951del	p.Y5317fs	0.28915663	913
3	*KMT2D*	NM_003482	chr12	49446461	TG	T	Frameshift deletion	c.1143delC	p.P381fs	0.42785235	596
4	*KMT2D*	NM_003482	chr12	49446347	C	T	Missense	c.G1258A	p.G420R	0.13	500
7	*KMT2D*	NM_003482	chr12	49431178	G	A	Nonsense	c.C9961T	p.R3321X	0.10652921	582
12	*KMT2D*	NM_003482	chr12	49425724	C	T	Missense	c.G12764A	p.G4255D	0.53038674	362
18	*KMT2D*	NM_003482	chr12	49433506	C	A	Splice site	c.8046+1G>T	NA	0.35344828	464
19	*KMT2D*	NM_003482	chr12	49447050	G	C	Nonsense	c.C894G	p.Y298X	0.336	1000
21	*KMT2D*	NM_003482	chr12	49433594	AC	A	Frameshift deletion	c.7958delG	p.G2653fs	0.33333333	708
25	*KMT2D*	NM_003482	chr12	49446816	C	A	Nonsense	c.G994T	p.E332X	0.28533686	757
30	*KMT2D*	NM_003482	chr12	49432209	T	C	Missense	c.A8930G	p.N2977S	0.42	900
33	*KMT2D*	NM_003482	chr12	49425041	G	C	Missense	c.C13447G	p.L4483V	0.07541899	716
37	*KMT2D*	NM_003482	chr12	49416114	C	T	Missense	c.G16361A	p.R5454Q	0.39480519	385
39	*KMT2D*	NM_003482	chr12	49431649	G	A	Missense	c.C9490T	p.R3164W	0.51688693	681
42	*KMT2D*	NM_003482	chr12	49416133	G	A	Nonsense	c.C16342T	p.R5448X	0.08938547	895
47	*KMT2D*	NM_003482	chr12	49431181	C	T	Missense	c.G9958A	p.A3320T	0.6089613	491
56	*KMT2D*	NM_003482	chr12	49425577	G	A	Missense	c.C12911T	p.P4304L	0.43333333	270
56	*KMT2D*	NM_003482	chr12	49440507	G	A	Nonsense	c.C4303T	p.Q1435X	0.39393939	726
60	*KMT2D*	NM_003482	chr12	49433394	G	A	Nonsense	c.C8053T	p.R2685X	0.42894057	387
63	*KMT2D*	NM_003482	chr12	49445511	C	T	Missense	c.G1955A	p.R652H	0.2605042	714
65	*KMT2D*	NM_003482	chr12	49448317	GTTCTCCTAGGTGGGCAGGT	G	Frameshift deletion	c.375_393del	p.T125fs	0.21984733	655
65	*KMT2D*	NM_003482	chr12	49448340	AGG	A	Frameshift deletion	c.369_370del	p.G123fs	0.23776224	572
65	*KMT2D*	NM_003482	chr12	49448348	AG	A	Frameshift deletion	c.362delC	p.P121fs	0.25168919	592
65	*KMT2D*	NM_003482	chr12	49421078	G	A	Nonsense	c.C14671T	p.Q4891X	0.43896104	385
65	*KMT2D*	NM_003482	chr12	49448338	T	A	Missense	c.A373T	p.T125S	0.2369338	574
70	*KMT2D*	NM_003482	chr12	49437166	T	C	Missense	c.A5513G	p.E1838G	0.35146444	717
73	*KMT2D*	NM_003482	chr12	49423183	C	A	Splice site	c.14075+1G>T	NA	0.49840256	626
81	*KMT2D*	NM_003482	chr12	49423182	A	T	Splice site	c.14075+2T>A	NA	0.40031397	637
85	*KMT2D*	NM_003482	chr12	49434758	TC	T	Frameshift deletion	c.6794delG	p.G2265fs	0.33030853	551
86	*KMT2D*	NM_003482	chr12	49426466	G	A	Missense	c.C12022T	p.H4008Y	0.46478873	710
87	*KMT2D*	NM_003482	chr12	49447416	G	A	Missense	c.C682T	p.R228C	0.45775536	793
91	*KMT2D*	NM_003482	chr12	49425900	C	G	Missense	c.G12588C	p.Q4196H	0.50073421	681
92	*KMT2D*	NM_003482	chr12	49431346	G	A	Nonsense	c.C9793T	p.Q3265X	0.27643312	785
93	*KMT2D*	NM_003482	chr12	49415855	AGAT	A	Nonframeshift deletion	c.16489_16491del	p.5497_54[illegible]	0.26526527	999
95	*KMT2D*	NM_003482	chr12	49447845	AG	A	Frameshift deletion	c.588delC	p.P196fs	0.60444444	675
95	*KMT2D*	NM_003482	chr12	49440143	A	C	Missense	c.T4483G	p.Y1495D	0.19247312	930
95	*KMT2D*	NM_003482	chr12	49440186	A	T	Nonsense	c.T4440A	p.C1480X	0.18577075	759
24	*MEF2B*	NM_001145785	chr19	19261528	A	T	Missense	c.T17A	p.I6N	0.25737898	847
28	*MEF2B*	NM_001145785	chr19	19260064	C	T	Missense	c.G229A	p.E77K	0.52867384	558
32	*MEF2B*	NM_001145785	chr19	19260165	A	C	Missense	c.T128G	p.I43R	0.26514132	743
39	*MEF2B*	NM_001145785	chr19	19260124	A	T	Missense	c.T169A	p.Y57N	0.22506394	782
43	*MEF2B*	NM_001145785	chr19	19256610	C	T	Missense	c.G1103A	p.R368Q	0.51546392	194
63	*MEF2B*	NM_001145785	chr19	19257101	CG	C	Frameshift deletion	c.861delC	p.P287fs	0.15976331	169
76	*MEF2B*	NM_001145785	chr19	19260237	A	AC	Frameshift insertion	c.55dupG	p.V19fs	0.12280702	570
85	*MEF2B*	NM_001145785	chr19	19260222	C	T	Missense	c.G71A	p.R24Q	0.34266667	750
37	*MEF2C*	NM_001193347	chr5	88018503	A	G	Missense	c.T1370C	p.I457T	0.59663866	476
67	*SETD2*	NM_014159	chr3	47129680	G	A	Nonsense	c.C5200T	p.Q1734X	0.25584112	856
84	*SETD2*	NM_014159	chr3	47098370	A	G	Missense	c.T6904C	p.C2302R	0.50554995	991
95	*SETD2*	NM_014159	chr3	47162915	C	T	Missense	c.G3211A	p.V1071I	0.88387824	887
12	*TET2*	NM_001127208	chr4	106157106	AAAAGCTC	A	Frameshift deletion	c.2008_2014del	p.K670fs	0.35198135	858
12	*TET2*	NM_001127208	chr4	106157969	T	TA	Frameshift insertion	c.2871dupA	p.L957fs	0.41271443	991
21	*TET2*	NM_001127208	chr4	106157786	T	G	Missense	c.T2687G	p.L896R	0.347	1000
29	*TET2*	NM_001127208	chr4	106156867	C	A	Missense	c.C1768A	p.L590I	0.49195171	994

30	*TET2*	NM_001127208	chr4	106196316	A	G	Missense	c.A4649G	p.H1550R	0.34700315	634
31	*TET2*	NM_001127208	chr4	106156069	C	T	Nonsense	c.C970T	p.Q324X	0.28	1000
31	*TET2*	NM_001127208	chr4	106157980	G	T	Nonsense	c.G2881T	p.E961X	0.367	1000
38	*TET2*	NM_001127208	chr4	106155363	TTTGCAAAATGGAGGAATAA	T	Frameshift deletion	c.265_283del	p.L89fs	0.15035461	705
47	*TET2*	NM_001127208	chr4	106196349	C	G	Missense	c.C4682G	p.S1561C	0.50180072	833
57	*TET2*	NM_001127208	chr4	106156791	GATTGA	G	Frameshift deletion	c.1693_1697del	p.I565fs	0.162	1000
57	*TET2*	NM_001127208	chr4	106156768	C	T	Nonsense	c.C1669T	p.Q557X	0.352	1000
63	*TET2*	NM_001127208	chr4	106197255	C	T	Missense	c.C5588T	p.A1863V	0.079	1000
64	*TET2*	NM_001127208	chr4	106157997	GC	G	Frameshift deletion	c.2899delC	p.Q967fs	0.452	1000
64	*TET2*	NM_001127208	chr4	106180783	T	TG	Frameshift insertion	c.3812dupG	p.C1271fs	0.44430538	799
82	*TET2*	NM_001127208	chr4	106158268	GA	G	Frameshift deletion	c.3170delA	:p.E1057fs,	0.26	1000
82	*TET2*	NM_001127208	chr4	106180783	T	TG	Frameshift insertion	c.3812dupG	p.C1271fs	0.2337376	907
5	*B2M*	NM_004048	chr15	45003764	T	G	Nonsense	c.T20G	p.L7X	0.46819338	393
14	*B2M*	NM_004048	chr15	45003780	ACT	A	Frameshift deletion	c.37_38del	p.L13fs	0.52141058	397
19	*B2M*	NM_004048	chr15	45007732	T	C	Missense	c.T179C	p.L60P	0.321	1000
19	*B2M*	NM_004048	chr15	45007753	A	T	Missense	c.A200T	p.E67V	0.329	1000
49	*B2M*	NM_004048	chr15	45003764	T	G	Nonsense	c.T20G	p.L7X	0.79545455	352
65	*B2M*	NM_004048	chr15	45003747	G	A	Missense	c.G3A	p.M1I	0.77118644	354
75	*B2M*	NM_004048	chr15	45003808	C	T	Nonsense	c.C64T	p.Q22X	0.82326622	447
80	*B2M*	NM_004048	chr15	45007620	G	A	Splice site	c.68-1G>A	NA	0.17467761	853
82	*B2M*	NM_004048	chr15	45003745	A	G	Missense	c.A1G	p.M1V	0.30107527	372
83	*B2M*	NM_004048	chr15	45003811	CGTGA	C	Splice site	c.67+4GTGA>-	NA	0.40751445	346
95	*B2M*	NM_004048	chr15	45003764	T	G	Nonsense	c.T20G	p.L7X	0.78271605	405
24	*CD58*	NM_001779	chr1	117086940	A	T	Nonsense	c.T357A	p.Y119X	0.23212192	853
24	*CD58*	NM_001779	chr1	117113523	A	G	Splice site	c.70+2T>C	NA	0.16608997	289
31	*CD58*	NM_001779	chr1	117087110	CT	C	Frameshift deletion	c.186delA	p.K62fs	0.107	1000
48	*CD58*	NM_001779	chr1	117113594	T	C	Missense	c.A1G	p.M1V	0.65644172	163
52	*CD58*	NM_001779	chr1	117087122	G	GT	Frameshift insertion	c.174dupA	p.Q59fs	0.359	1000
83	*CD58*	NM_001779	chr1	117078761	G	A	Nonsense	c.C454T	p.R152X	0.48427673	636
92	*CD58*	NM_001779	chr1	117113592	C	T	Missense	c.G3A	p.M1I	0.31794872	195
4	*CD70*	NM_001252	chr19	6590114	C	T	Missense	c.G196A	p.G66R	0.14448669	526
8	*CD70*	NM_001252	chr19	6590118	GTGATTC	G	Nonframeshift deletion	c.186_191del	p.62_64del	0.3121547	362
16	*CD70*	NM_001252	chr19	6586126	G	A	Nonsense	c.C487T	p.R163X	0.16018307	437
48	*CD70*	NM_001252	chr19	6590113	C	T	Splice site	c.196+1G>A	NA	0.77677419	775
63	*CD70*	NM_001252	chr19	6586119	T	G	Missense	c.A494C	p.D165A	0.55887522	569
63	*CD70*	NM_001252	chr19	6590147	A	T	Missense	c.T163A	p.W55R	0.09179416	719
67	*CD70*	NM_001252	chr19	6590114	C	T	Missense	c.G196A	p.G66R	0.28915663	415
68	*CD70*	NM_001252	chr19	6590114	C	T	Missense	c.G196A	p.G66R	0.27444254	583
71	*CD70*	NM_001252	chr19	6590890	CGAAGCGCTGG	C	Frameshift deletion	c.114_123del	p.I38fs	0.118	1000
71	*CD70*	NM_001252	chr19	6590902	T	TC	Frameshift insertion	c.111_112insG	p.I38fs	0.116	1000
71	*CD70*	NM_001252	chr19	6590903	G	C	Missense	c.C111G	p.C37W	0.11711712	999
74	*CD70*	NM_001252	chr19	6590148	C	T	Splice site	c.163-1G>A	NA	0.71904762	840
75	*CD70*	NM_001252	chr19	6590913	A	C	Missense	c.T101G	p.L34R	0.86379115	881
83	*CD70*	NM_001252	chr19	6591012	A	G	Missense	c.T2C	p.M1T	0.68410853	516
9	*CIITA*	NM_001286402	chr16	11001303	A	G	Missense	c.A1957G	p.S653G	0.58188153	861
16	*CIITA*	NM_001286402	chr16	11000835	C	T	Missense	c.C1489T	p.L497F	0.53640777	824
24	*CIITA*	NM_001286402	chr16	10971232	G	GC	Frameshift insertion	c.46dupC	p.E15fs	0.18193384	786
50	*CIITA*	NM_001286402	chr16	10971240	G	A	Splice site	c.52+1G>A	NA	0.29232996	691
61	*CIITA*	NM_001286402	chr16	10989149	G	C	Missense	c.G63C	p.Q21H	0.53813559	472
63	*CIITA*	NM_001286402	chr16	10992583	AT	A	Frameshift deletion	c.353delT	p.I118fs	0.2457293	761
67	*CIITA*	NM_001286402	chr16	10995936	C	T	Missense	c.C526T	p.P176S	0.46511628	516
72	*CIITA*	NM_001286402	chr16	10971239	G	A	Missense	c.G52A	p.G18S	0.46746348	753
72	*CIITA*	NM_001286402	chr16	11012358	C	T	Missense	c.C3127T	p.L1043F	0.47674419	430
84	*CIITA*	NM_001286402	chr16	11001533	C	G	Missense	c.C2187G	p.I729M	0.47452229	942
93	*CIITA*	NM_001286402	chr16	11001533	C	G	Missense	c.C2187G	p.I729M	0.47982063	892

95	*CIITA*	NM_001286402	chr16	10995372	CT	C	Frameshift deletion	c.442delT	p.F148fs	0.27720739	487
95	*CIITA*	NM_001286402	chr16	11000499	A	T	Missense	c.A1153T	p.T385S	0.215	600
96	*CIITA*	NM_001286402	chr16	11000547	G	A	Missense	c.G1201A	p.V401M	0.62441315	852
7	*TNFRSF14*	NM_003820	chr1	2488123	G	A	Nonsense	c.G20A	p.W7X	0.12709832	417
8	*TNFRSF14*	NM_003820	chr1	2489232	A	G	Missense	c.A137G	p.E46G	0.73255814	172
19	*TNFRSF14*	NM_003820	chr1	2489189	G	GC	Frameshift insertion	c.95dupC	p.A32fs	0.52538071	394
24	*TNFRSF14*	NM_003820	chr1	2489886	C	T	Nonsense	c.C283T	p.Q95X	0.26760563	568
52	*TNFRSF14*	NM_003820	chr1	2488138	G	A	Nonsense	c.G35A	p.W12X	0.55514706	272
68	*TNFRSF14*	NM_003820	chr1	2489899	G	C	Missense	c.G296C	p.C99S	0.16078431	510
68	*TNFRSF14*	NM_003820	chr1	2492068	G	A	Missense	c.G466A	p.E156K	0.38607595	316
70	*TNFRSF14*	NM_003820	chr1	2488138	G	A	Nonsense	c.G35A	p.W12X	0.37898089	314
82	*TNFRSF14*	NM_003820	chr1	2488165	T	A	Nonsense	c.T62A	p.L21X	0.54977376	442
83	*TNFRSF14*	NM_003820	chr1	2492096	G	GC	Frameshift insertion	c.495dupC	p.C165fs	0.60042283	473
85	*TNFRSF14*	NM_003820	chr1	2493240	G	T	Missense	c.G680T	p.R227I	0.69811321	742

Abbreviations: AA, amino acid; NA, not available.

Table S5 Potential therapeutic targets based on gene mutations in DLBCL

Gene	Therapeutic agent
CD79A/CD79B	BTK inhibitors (ibrutinib), SYK inhibitors (fostamatinib, entospletinib), PKC inhibitors (sotrastaurin)
MYD88	IRAK1/4 inhibitors
EZH2	EZH2 inhibitors (tazemetostat, GSK126)
CARD11	MALT1 inhibitors
CREBBP/EP300	HDAC inhibitors (panobinostat, vorinostat, mocetinostat, abexinostat)
TNFAIP3	Proteasome inhibitors (bortezomib, carfilzomib)
JAK3	JAK inhibitors
SOCS1	JAK inhibitors
STAT6	STAT inhibitors
NOTCH1	Notch inhibitors
NOTCH2	Notch inhibitors
SPEN	Notch inhibitors

Table S6 Univariate survival analysis for 41 genes in patients with DLBCL

	All patients		R-CHOP group		All patients		R-CHOP group	
Gene	*P* (PFS)	FDR	*P* (PFS)	FDR	*P* (OS)	FDR	*P* (OS)	FDR
KMT2D	0.014	0.177	0.032	0.395	0.028	0.261	0.046	0.288
SOCS1	0.113	0.662	0.271	0.803	0.063	0.441	0.141	0.588
MYD88	0.061	0.464	0.011	0.204	0.172	0.688	0.024	0.200
MYD88 L265P	0.007	0.133	0.000183	**0.006771**	0.088	0.481	0.009	0.113
CD79B	0.335	0.792	0.185	0.803	0.944	0.979	0.632	0.830
PIM1	0.883	0.963	0.679	0.837	0.803	0.979	0.527	0.830
BTG1	0.813	0.963	0.797	0.894	0.512	0.979	0.329	0.830
SPEN	0.679	0.963	0.471	0.803	0.775	0.979	0.500	0.830
TBL1XR1	0.438	0.876	0.178	0.803	0.919	0.979	0.370	0.830
SGK1	0.810	0.963	0.437	0.803	0.674	0.979	0.947	0.986
HIST1H1E	0.255	0.792	0.973	0.973	0.474	0.979	0.985	0.986
CREBBP	0.825	0.963	0.706	0.843	NA	NA	NA	NA
TET2	0.369	0.792	0.507	0.803	NA	NA	NA	NA
KLHL6	NA	NA	NA	NA	NA	NA	NA	NA
TNFRSF14	0.141	0.670	0.401	0.803	NA	NA	NA	NA
B2M	0.920	0.963	0.784	0.894	0.929	0.979	0.255	0.830
FAS	0.320	0.792	0.436	0.803	NA	NA	NA	NA
TP53	0.007	0.133	0.194	0.803	0.024	0.261	0.428	0.830
KMT2C	0.963	0.963	0.648	0.837	0.854	0.979	0.440	0.830
CIITA	0.354	0.792	0.453	0.803	0.448	0.979	0.594	0.830
CARD11	0.677	0.963	0.514	0.803	0.614	0.979	0.540	0.830
IRF4	0.632	0.963	0.670	0.837	0.103	0.481	0.126	0.588
NOTCH2	0.708	0.963	NA	NA	0.894	0.979	NA	NA
POU2F2	0.122	0.662	0.382	0.803	0.483	0.979	NA	NA
HIST1H1C	0.222	0.792	0.167	0.803	0.405	0.979	0.269	0.830
MEF2B	0.365	0.792	0.480	0.803	NA	NA	NA	NA
NOTCH1	0.661	0.963	0.325	0.803	0.860	0.979	0.664	0.830
GNA13	NA	NA	NA	NA	NA	NA	NA	NA
STAT3	0.225	0.792	0.334	0.803	NA	NA	NA	NA
STAT6	0.817	0.963	0.556	0.803	0.523	0.979	NA	NA
ETV6	0.910	0.963	0.564	0.803	0.436	0.979	0.986	0.986
CD70	0.375	0.792	0.556	0.803	0.692	0.979	0.973	0.986
BCL6	0.946	0.963	0.417	0.803	NA	NA	NA	NA
ACTB	NA	NA	NA	NA	NA	NA	NA	NA
CD58	0.293	0.792	0.617	0.837	NA	NA	NA	NA
ARID1A	0.489	0.929	0.927	0.957	0.737	0.979	0.597	0.830
FOXO1	0.957	0.963	0.931	0.957	NA	NA	NA	NA
PRDM1	0.608	0.963	0.846	0.921	NA	NA	NA	NA
PTEN	0.811	0.963	0.432	0.803	0.995	0.995	0.660	0.830
BCL2	0.023	0.219	0.161	0.803	0.000306	**0.008568**	0.005	0.113
DDX3X	NA	NA	NA	NA	NA	NA	NA	NA
EP300	0.686	0.963	0.349	0.803	0.793	0.979	0.944	0.986

Abbreviation: R-CHOP, rituximab, cyclophosphamide, doxorubicin, vincristine, and prednisone; PFS, progression-free survival; OS, overall survival; FDR, false discovery rate; NA, not available.
